# Transformation of Natural Resin Resina Draconis to 3D Functionalized Fibrous Scaffolds for Efficient Chronic Wound Healing

**DOI:** 10.1002/adhm.202401105

**Published:** 2024-06-25

**Authors:** Shijie Guo, Pengyu Wang, Yu Sun, Can Cao, Junwei Gao, Shihao Hong, Ning Li, Ruodan Xu

**Affiliations:** ^1^ Department of Biomedical Engineering and Technology Institute of Basic Theory for Chinese Medicine China Academy of Chinese Medical Sciences Beijing 100700 China; ^2^ Guang'anmen Hospital China Academy of Chinese Medical Sciences Beijing 100053 China

**Keywords:** 3D structure, bioactive wound dressing, chronic wound healing, Resina draconis, wet electrospinning

## Abstract

Chronic wound healing is a major challenge in clinical practice. Secondary dressing damage and antibiotic resistance are the main obstacles for traditional wound dressings. Resina draconis (RD), a natural resin traditionally used in powder form for wound care, is now considered unsuitable due to the lack of gas permeability and moist environment required for wound healing. Here, RD is incorporated in situ by constructing a 3D coiled fibrous scaffold with polycaprolactone/polyethylene oxide. Due to the high porosity of 3D scaffold, the RD‐3D dressings have a favorable swelling capacity, providing permeability and moisture for wound repair. Meanwhile, the transformation of RD powder into 3D dressings fully demonstrates capabilities of RD in rapid hemostasis, bactericidal, and inflammation‐regulating activities. In vivo evaluations using pressure ulcer and infected wound models confirm the high efficacy of RD‐3D dressing in early wound healing, particularly beneficial in the infected wound model compared to recombinant bovine FGF‐basic. Further biological analysis shows that resveratrol, loureirin A, and loureirin B, as potentially bioactive components of RD, individually contribute to different aspects of wound healing. Collectively, RD‐3D integrated dressings represent a simple, cost‐effective, and safe approach to wound healing, providing an alternative therapy for translating medical dressings from bench to bedside.

## Graphical abstract

A novel bioactive dressing with coiled fibers and nanopores in microstructure is fabricated by integrating traditional Chinese medicine Resina draconis into the 3D polycaprolacton/polyethylene oxide scaffold using wet electrospinning for chronic wound management. This dressing shows excellent properties for a moist wound microenvironment, offering cell‐proliferative, hemostatic, antibacterial, and inflammation‐regulating features for pressure ulcers and infected wounds.

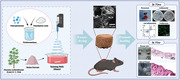

## 1 Introduction

Chronic wounds are wounds that do not heal according to normal physiological processes and often become life‐threatening over time. Etiologically, chronic wounds are classified as infected wounds, pressure ulcers, diabetic ulcers, and vascular ulcers.^[^ ^1^ ^]^ Patients with chronic wounds may suffer from local swelling, redness, and pain, and unhealed ulcers and infections can quickly become serious, leading to amputation, sepsis, and even death. Such serious outcomes substantially limit patients’ activities and reduce their quality of life.^[^ ^2^, ^3^ ^]^ The incidence of chronic wounds is growing rapidly like a “silent epidemic”,^[^ ^4^ ^]^ which is not only a major public health concern, but also has a profound financial and psychological impact, resulting in a heavy socioeconomic burden.^[^ ^5^, ^6^, ^7^ ^]^

The management of chronic wounds relies on frequent changes of medical dressings, which are an essential part of wound care as they generally prevent trauma, keep out dirt and germs, absorb exudate, and maintain a moist microenvironment to promote healing.^[^ ^8^ ^]^ As first choice dressings, commonly used dressings such as gauze, bandages, and cotton wool are recognized as providing a protective barrier. However, these dressings dry out quickly and easily stick to the wound, leading to re‐injury and wound deterioration. Various tactics have been used to overcome these problems, of which electrospinning to produce nano/microscale fibers has attracted dramatic attention. Since fiber‐based dressings have a high surface area with greater drug loading capacity, controllable porosity for efficient oxygen exchange, and limited pore size against exogenous microbial invasion,^[^ ^9^ ^]^ electrospun mats are considered one of the ideal dressings for wound healing. However, conventional electrospinning usually produces densely packed nanofiber mats in 2D rather than 3D porous structures, which may compromise gas permeability and their absorption potential for wound exudates.^[^ ^10^, ^11^ ^]^ Although numerous strategies for 3D electrospun structures have been developed, such as gas foaming, template‐assisted electrospinning, and multilayer electrospinning, each technology has its limitations. For instance, gas foaming cannot be used to realize tunable 3D geometries and is difficult to use as a carrier for drugs. Moreover, template‐assisted collection usually requires a specific complex collector, and the resulting product has weak mechanical properties. In addition, the 3D scaffold fabricated by multilayer electrospinning usually consists of a single‐layer membrane and hence has an inflexible shape. Therefore, compared to the most commonly used gas‐forming, template, and multilayer techniques, wet electrospinning is able to produce fibers with increased interfiber space and uniform pore size, a condition more desirable for wound healing. Despite its structural advantages, this technique does not require postprocessing steps such as salt leaching or template removal, making it convenient to operate.

In terms of loaded therapeutic agents, dressings containing bioactive components in the form of antibiotics, growth factors, and metal elements have been designed and developed for rapid hemostasis, antibacterial and immunoregulatory purposes.^[^ ^12^ ^]^ It is estimated that almost 50% of commercially available wound dressings contain antibiotics, the overuse of which is associated with a number of risks including allergy, toxicity, bacterial dysbiosis, and resistance.^[^ ^13^ ^]^ As an alternative compelling strategy, natural herbs have been applied for centuries to promote wound healing due to their multitarget effects, reduced side effects, and ability to promote the natural healing process.^[^ ^14^ ^]^ Resina draconis (RD) is a resin obtained from *Dracaena cochinchinensis* (Lour.) S. C. Chen (Yunnan, China). As a traditional Chinese medicine, RD has been used in China for more than 1500 years for a variety of clinical conditions, including but not limited to wound care. The multiple pharmacological effects of RD in promoting wound healing have been demonstrated in a wide range of wound types, including traumatic soft tissue defects, pressure ulcers, and longstanding nonhealing wounds.^[^ ^15^, ^16^, ^17^ ^]^ Clinical studies have confirmed the efficacy of RD in hemostasis, analgesia, anti‐inflammation, and angiogenesis, the biological events that contribute to the healing process, demonstrating the wide applicability and broad therapeutic potential of RD.^[^ ^18^, ^19^, ^20^, ^21^ ^]^ However, with advances in the understanding of wound healing, RD in its original powder form needs to be reconsidered. It has been found that topical application of the powder does not promote oxygen exchange or maintain a moist healing environment necessary for proper wound healing, and sometimes an excessively dry environment can slow down the healing process.^[^ ^22^ ^]^ In addition, in the presence of wound secretions, powdered medications can clump and adhere to the wound, causing secondary injury during dressing changes.^[^ ^23^ ^]^ Although a number of topical RD formulations have been developed, the inefficient absorption of topical RD, such as in ointments and dispersions, due to the poor water solubility of RD has further challenged the use of RD. One strategy to address the solubility and bioavailability of poorly soluble drugs is again the electrospinning technique. In addition to enabling the production of fibrous materials with high porosity and favorable absorption potential as mentioned above, electrospinning could successfully achieve solubility of insoluble drugs by solid dispersion, whereby a drug such as RD is converted to a highly dispersed microcrystalline or amorphous state in another solid fibrous carrier material. At present, the potential of electrospun RD as a wound dressing remains to be investigated.

In the current study, we developed a simple and easily reproducible RD‐loaded polycaprolactone (PCL)/polyethylene oxide (PEO) dressing by integrating RD with wet electrospinning. The combination of hydrophobic PCL and hydrophilic PEO is a common strategy in electrospinning, as each material has unique advantages and is compatible with each other to provide complementary performance, particularly in wound healing. For example, by taking advantage of the slow‐release properties of PCL and the rapid dissolution and release properties of PEO, it is possible to design drug delivery systems with biphasic mechanisms to meet the needs of different stages of wound healing. Moreover, the hydrophobicity of PCL combined with the water solubility and biocompatibility of PEO can result in composites with desirable bioactivity and moisture environment to promote cell adhesion and growth required for wound healing. As designed, the 3D‐structured PCL/PEO dressing produced in this study exhibited high porosity, absorbency and excellent water retention properties. In addition, regarding the bioactivity of RD, three identified active components, including loureirin A, resveratrol, and loureirin B could be efficiently encapsulated in 3D PCL/PEO and then triggered for release.^[^ ^24^, ^25^, ^26^, ^27^, ^28^, ^29^, ^30^, ^31^ ^]^ The loading of RD via van der Waals forces endowed the 3D RD‐loaded PCL/PEO dressings (RD‐3D) with both hemostasis and antibacterial properties. In two chronic wound models of mouse, including infected wounds and pressure ulcers, RD‐3D dressings effectively ameliorated infection and accelerated full‐thickness wound regeneration with no detectable adverse effects. Further detailed analysis revealed that the therapeutic efficacy of RD‐3D fibrous dressings could be attributed to 1) the vascularizing and hemostatic activity of loureirin A; 2) the antibacterial potentials of resveratrol; and 3) the ability of loureirin A, loureirin B, and resveratrol to accelerate fibroblast proliferation and migration. Thus, loading of RD into a 3D dressing overcame the low bioavailability of RD and empowered the 3D fibers with wound repair bioactivities. The conversion of natural herbs or their bioactive components into 3D medical fibers provides a more insightful understanding of the mechanisms of RD in the management of chronic wounds and offers a new methodology for the next generation of wound dressings.

## 2 Results and Discussion

### 2.1 The Coiled and Porous Structure of 3D and RD‐3D Dressings

**Figure 1:**
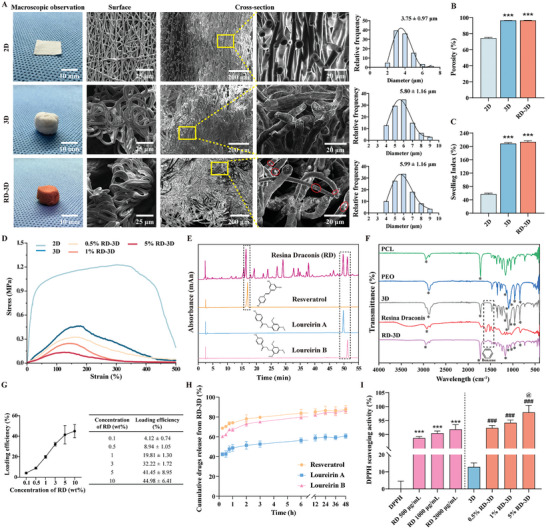
Characterizations of RD‐3D fibrous dressings. A) Macroscopic observations and SEM images of the surfaces and cross‐sections of 2D (top panels), 3D (middle panels), and RD‐3D (bottom panels) dressings, with corresponding diameter distributions (right). SEM images in the right column represent magnifications of the corresponding dashed frames in yellow. Red circles indicate RD particles. B) The percentage of porosity and C) swelling index of 2D, 3D, and RD‐3D. ***p < 0.005 versus 2D. D) Stress–strain curves of 2D, 3D, and RD‐3D. E) RP‐HPLC analysis of RD with its major components resveratrol, loureirin A, and loureirin B as reference standards. Black dashed frames: characteristic peaks of each condition. F) FTIR spectra of PCL, PEO, 3D, RD, and RD‐3D. Asterisks (*) indicate characteristic peaks of the samples listed in Table S2 (Supporting Information). Black dashed frames: characteristic peaks of the benzene ring. G) Drug loading efficiencies of RD‐3D were collected from 0.1, 0.5, 1, 3, 5, and 10 (wt%) initial RD ethanol solution, with absolute values on the right panel. H) In vitro cumulative drug release profiles of resveratrol, loureirin A, and loureirin B from RD‐3D within 48 h. I) Antioxidant capacities of RD, 3D, and RD‐3D at different concentrations were evaluated by DPPH radical scavenging assays. ***p < 0.005 versus DPPH; ^###^ *p* < 0.005 versus 3D; ^@^ *p* < 0.05 versus 0.5% RD‐3D. Bars represent mean ± SEM of three independent assays.

To obtain the RD‐incorporated 3D porous structure by wet electrospinning, the optimal 15% PCL/PEO with a composition ratio of 95:5 (w/w) was determined by considering a combination of five parameters, including surface smoothness, fiber homogeneity, high porosity, excellent water retention, and amphipathic property (Figure S1A–C, Supporting Information). The application of conventional electrospinning using a solid collector resulted in a thin white paper‐like membrane composed of 2D fibers with an average diameter of 3.75 ± 0.97 µm (**Figure** **1**A, top panels). In contrast to the randomly organized fibers in the flat 2D membrane, the surface morphology of the 3D fibers and RD‐loaded 3D fibers prepared by single‐step wet electrospinning showed a coiled structure under SEM (Figure 1A). The average diameters were measured to be 5.80 ± 1.16 and 5.99 ± 1.16 µm for 3D and RD‐3D, respectively, which tended to be larger than 2D fibers (3.75 ± 0.97 µm). The formation of coiled fibers in wet electrospinning can be attributed to the combined effects of the tensile and normal components of the electric force on the electrospun jet, as well as the surface tension of the nonsolvent bath. In the current study, firstly, the fine streams from wet electrospinning are not usually solidified directly in air but are guided to anhydrous ethanol, where phase separation occurs rapidly in the primary liquid stream due to solvent/nonsolvent interaction, resulting in polymer precipitation and solidification into fibers. When a small elastic or viscoelastic electrospun structure comes into contact with a liquid surface, capillary forces can cause bending or wrinkling. This can lead to compressive forces strong enough to induce buckling, resulting in higher frequency, low‐amplitude coiling. Secondly, when using anhydrous ethanol as a coagulation bath, the anhydrous ethanol can accelerate the curing process of the fibers, particularly at the fiber surface. Rapid curing locks in morphological changes in the fibers caused by surface tension, internal stresses, and so on, during the dynamic curing process. When the internal elastic recovery forces do not fully compensate for these external deformations, permanent curling of the fibers can occur. Thirdly, the anhydrous ethanol coagulation bath in this study was stirred continuously, and the shear forces caused by the flow may cause the fibers to twist or bend before solidification, and during the curing process these deformations may be fixed to form a curled structure. Therefore, the formation of coiled fibers of 3D and RD‐3D is mainly triggered by the continuously stirred anhydrous ethanol bath.

To better observe the microstructures inside the fibers, cross‐sections of the specimen are prepared. In the 2D membrane, solid cylindrical fibers were observed, tightly packed in a lamellar organization. In contrast to the 2D structure, a large number of nanopores were found in the cross‐sections of the 3D and RD‐3D fibers. By further quantifying the porosity of the fibers, we verified that the 3D and RD‐3D fibers were more porous than the 2D membrane, with porosities of 96.46 ± 0.42%, 96.52 ± 0.42%, and 74.36 ± 1.76% for 3D, RD‐3D and 2D structures, respectively (Figure 1B). Consistent with the increased porosity, the 3D fibers had a much higher swelling index, meaning that the 3D fibrous scaffolds can absorb more moisture than 2D fibrous membranes (Figure 1C). Although the high porosity of 3D and RD‐3D dressings is favorable for gas permeability and fluid management, it may sacrifice the mechanical properties of the dressing, including tensile strength and elongation potential. Generally, an increase in porosity is often accompanied by a decrease in scaffold density and weak interpore connections, which directly affects the tensile strength and maximum tensile strain of the dressings. As expected, the mechanical properties, including tensile strength, percent elongation at break and Young's modulus, of the 3D dressings were lower than those of the 2D mats (0.44 ± 0.02 MPa vs 1.18 ± 0.10 MPa; 208.20 ± 15.26% vs 331.00 ± 11.12%; 0.35 ± 0.02 MPa vs 1.18 ± 0.10 MPa), indicating lower stiffness and flexibility of the 3D and RD‐3D dressings. When the concentration of RD in the 3D dressings was increased from 0% to 5%, the mechanical properties of the 3D dressings were further reduced. In this case, the grafting of RD onto nanofibers resulted in a weakening of the mechanical behavior (Figure 1D and **Table** **1**). Given that the Young's modulus range for human skin is 0.1–2 MPa,^[^ ^34^ ^]^ the strength and flexibility of dressings for wound healing may vary depending on the specific application. Here, the 3D and RD‐3D may be more appropriate for wounds in areas of the body with low mobility.

**Table 1:** The mechanical characteristics of 2D, 3D, RD‐3D (Mean ± SEM, *n* = 3).

Samples	Tensile strength [MPa]	Elongation at break [%]	Young's modulus [MPa]
2D	1.18 ± 0.10	331.00 ± 11.12	1.18 ± 0.10
3D	0.44 ± 0.02	208.20 ± 15.26	0.35 ± 0.02
0.5% RD‐3D	0.30 ± 0.02	178.70 ± 5.10	0.21 ± 0.01
1% RD‐3D	0.21 ± 0.02	167.70 ± 7.92	0.15 ± 0.01
5% RD‐3D	0.14 ± 0.01	151.00 ± 14.74	0.12 ± 0.01

### 2.2 Incorporation and Distribution of RD in RD‐3D Dressings

In addition to the presence of porous nanostructure in 3D and RD‐3D dressings by SEM, the number of nanopores was dramatically reduced both on the surface and in the cross‐sections of RD‐3D compared to 3D fibers (Figure 1A). Meanwhile, drug particles were observed on the surface of RD‐3D fibers, indicating that RD is dispersed both inside and on the surface of the fibers. Since RD is dissolved in stirred anhydrous ethanol during wet electrospinning, the electrospun RD molecules can either be embedded inside the fiber, dispersed in the fiber structure in the coagulation bath or absorbed onto the surface of the already formed electrostatically spun fibers. To confirm that RD was successfully loaded into the PCL/PCO scaffolds, three principal bioactive components of RD, namely resveratrol, loureirin A, and loureirin B, were then evaluated by both reversed‐phase high‐performance liquid chromatography (RP‐HPLC) (Figure 1E and Figure S2, Supporting Information) and Fourier transform infrared (FTIR) spectroscopy (Figure 1F and Table S2, Supporting Information). As shown in Figure 1E, by comparing the chemical profiles of RD‐3D with the reference standards of resveratrol, loureirin A, and loureirin B, clear peaks corresponding to the three components could be recognized.

In addition to RP‐HPLC, FTIR further identifies chemical bonds in a molecule. In agreement with previous reports on PCL and PEO, typical FTIR spectra of PEL/PEO could be recorded.^[^ ^35^, ^36^ ^]^ As shown in Figure 1F, the characteristic infrared bands of PCL at 2949, 2868, 1724, 1240, and 1184 cm^−1^ were assigned to asymmetric CH_2_ stretching, symmetric CH_2_ stretching, C─O stretching in crystalline phases, asymmetric C─O─C stretching, and symmetric C─O─C stretching, respectively. Similarly, the FTIR spectra of pure PEO obtained at 2880 and 1095 cm^−1^ were attributed to the typical stretching vibration of either the C─H group or the C─O─C group, respectively. In the spectrum of 3D scaffolds, we observed the typical FTIR spectra of both PCL and PEO, including asymmetric and symmetric CH_2_ stretching at 2945 and 2880 cm^−1^, and symmetric stretching vibration peak of C═O at 1724 cm^−1^. For RD alone, absorption peaks were observed around 1450–1600 cm^−1^, 2870 and 2950 cm^−1^ defined as aromatic C═C stretching, and C─H stretching, which could be explained by the stretching vibration of the benzene ring of aromatic components in RD.^[^ ^37^, ^38^ ^]^ For RD‐loaded 3D scaffolds, the specific peaks of RD, especially aromatic ring stretching between 1450 and 1600 cm^−1^, were present in the spectra of 3D scaffolds. This confirmed the successful incorporation of RD into the 3D scaffolds. Furthermore, we observed a slight shift in the absorption frequency in the FTIR spectra of RD‐loaded 3D scaffolds, possibly due to dipolar interactions, interfacial effects, and surface amorphousness. Importantly, no new peaks were observed, indicating that the chemical structure of RD remained unchanged during the loading process.

To further elucidate the loading efficiency of RD into 3D fibers, different concentrations of RD were electrospun to PCL/PEO. As depicted in Figure 1G, the loading efficiency of RD improved as the amount of RD in ethanol increased from 0.1% to 10% (w/w), with the best linear relationship occurred between 0.5% and 5% (w/w) of RD. More specifically, the loading of 0.5%, 1%, and 5% RD into 3D mats corresponds to 8.94 ± 1.05%, 19.81 ± 1.30%, and 41.45 ± 8.95% loading efficiency of RD, respectively, representing a twofold relationship. Hence, we selected 0.5%, 1%, and 5% RD for the following evaluations.

### 2.3 Incorporated RD Releases in a Biphasic Profile and Retains RD's Bioactivities

To better understand the therapeutic potential of RD, we evaluated the dynamic release of resveratrol, loureirin A, and loureirin B using 5% RD‐3D. As evidenced in Figure 1H, the release profile of RD‐3D showed a biphasic behavior, starting with a burst release followed by a sustained release. In particular, ≈56.3% of loureirin A was released from RD‐3D within 6 h, followed by a slow liberation to 60.71% at the end of 48 h, and a much faster release of resveratrol and loureirin B within 6 h was recorded at 83.92% and 79.84%, which increased steadily to 87.69% and 86.54% at the end of 48 h. The burst release of RD from 3D‐RD could be due to the detachment of RD from the surface, while the sustained phase is partially supported by the RD incorporated into the fibers. The benefits of biphasic drug release in wound healing have been recognized as facilitating a rapid onset of action at the wound site whilst maintaining a consistent drug dose throughout the wound healing process.

To further demonstrate that RD incorporated into a 3D scaffold does not alter the bioactivities of RD, we investigated the antioxidant function of RD, which has been shown to contribute to the healing process.^[^ ^39^ ^]^ As shown in Figure 1I, we tested the DPPH free radical scavenging activity using 0.5%, 1%, and 5% RD‐3D (0.5%, 1%, and 5% RD in the coagulation bath during the preparation of RD‐3D), corresponding to 500, 1000, and 2000 µg mL^−1^ loaded RD, respectively. When comparing the DPPH scavenging ability of RD with that of RD‐3D, we found that the antioxidant activity for RD alone was 88.71% ± 0.60%, 90.45% ± 0.82%, and 91.85% ± 1.72% for 500, 1000, and 2000 µg mL^−1^ RD, respectively, which was moderately increased to 92.35% ± 0.91%, 94.20% ± 1.03%, and 97.98% ± 2.47% for 0.5%, 1%, and 5% RD‐3D, respectively. Our results suggest that the basic bioactivity of RD remains intact when incorporated into a 3D scaffold and acts synergistically with PCL/PEO in terms of antioxidant activity.

### 2.4 RD‐3D Fibrous Dressing Has Favorable In Vitro Biocompatibility

**Figure 2:**
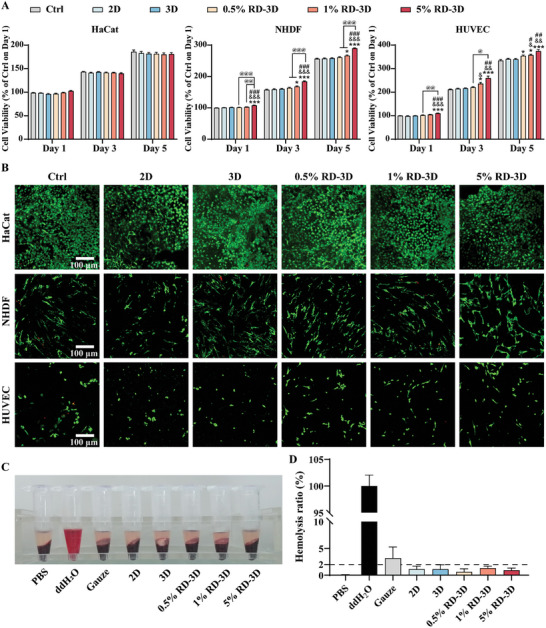
RD‐3D fibrous dressing has favorable in vitro biocompatibility. A) The respective cell viability of HaCat, NHDF, and HUVEC after incubation with soaking solutions of 2D, 3D, 0.5% RD‐3D, 1% RD‐3D, or 5% RD‐3D fibrous dressings for 1, 3, and 5 d (results normalized to untreated control on day 1; *p < 0.05, ***p < 0.005 versus Ctrl on the same day; ^&^ *p* < 0.05, ^&&^ *p* < 0.01, ^&&&^ *p* < 0.005 versus 2D on the same day; ^#^ *p* < 0.05, ^##^ *p* < 0.01, ^###^ *p* < 0.005 versus 3D on the same day; ^@^ *p* < 0.05, ^@@^ *p* < 0.01, ^@@@^ *p* < 0.005 as indicated). B) Fluorescence micrograph of the live/dead cell assay performed on day 5 (green cells are live, red cells are dead). Scale bar: 100 µm. C) Photographs from the hemocompatibility assay of gauze, 2D, 3D, 0.5% RD‐3D, 1% RD‐3D, and 5% RD‐3D. PBS and ddH_2_O were used as the negative and positive controls, respectively. D) The hemolysis ratio was quantified using samples from (C). Bars represent mean ± SEM of 3 independent experiments.

During the wound healing process, several factors contribute to tissue repair, including re‐epithelialization by keratinocytes, collagen regeneration by fibroblasts, and angiogenesis induced by vascular endothelial cells.^[^ ^40^ ^]^ To ensure the biocompatibility of the fibrous dressings, cytotoxicity and live/dead staining were performed. As shown in **Figure** **2**A, no cytotoxic effects were observed in any of the cells tested, including human keratinocytes (HaCat), normal human dermal fibroblasts (NHDF), and human umbilical vein endothelial cells (HUVEC), supporting a good biocompatibility of the fibers and RD‐incorporated dressings. In all cells tested, 5% RD‐3D significantly induced cell proliferation of NHDF and HUVEC from day 1, and both 1% and 5% RD‐3D fibers remarkably triggered the growth of NHDF and HUVEC on day 3 and day 5. Live/dead cell staining on day 5 verified their cytocompatibility, with no dead cells staining in red (Figure 2B).

To evaluate the blood compatibility of RD‐3D, the assessment of hemolysis is critical as damage is often induced by biomaterials and/or their extracts coming into direct or indirect contact with blood cells in vitro.^[^ ^41^ ^]^ In Figure 2C,D, the visible red aqueous phase was examined and quantitatively compared with the negative control PBS (0% hemolysis) and the positive control ddH_2_O (100% hemolysis). None of the dressings tested showed any apparent hemolytic activity at 37 °C. According to the Standard Practice for Assessment of Hemolytic Properties of Materials ASTM F756‐17, materials can be further classified into three types based on the degree of hemolysis: nonhemolytic (hemolysis < 2%), slightly hemolytic (hemolysis between 2% and 5%), and hemolytic (hemolysis ≥ 5%).^[^ ^42^ ^]^ We found that the gauze caused mild hemolysis with a hemolysis ratio of 3.16 ± 2.12%, whereas the hemolysis ratios of 2D (1.11 ± 0.58%), 3D (1.11 ± 0.85%), 0.5% RD‐3D (1.60 ± 0.57%), 1% RD‐3D (1.32 ± 0.36%), and 5% RD‐3D (0.94 ± 0.40%) were kept below 2% and classified as non‐hemolytic (Figure 2D). Therefore, the RD‐3D fibrous dressings are safe and suitable for use as medical biomaterials.

### 2.5 RD‐3D Fibrous Dressing with Effective Hemostasis

**Figure 3:**
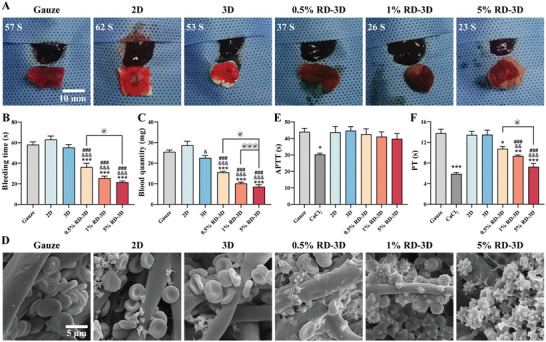
RD‐3D fibrous dressing with effective hemostasis. A) Macroscopic images of mouse liver at the time of in vivo hemostasis after treatment with gauze, 2D, 3D, 0.5% RD‐3D, 1% RD‐3D, or 5% RD‐3D. Scale bar: 10 mm. B,C) Bleeding time and blood volume in mouse liver hemorrhage models from (A). ***p < 0.005 versus Gauze; ^&^ *p* < 0.05, ^&&&^ *p* < 0.005 versus 2D; ^###^ *p* < 0.005 versus 3D; ^@^ *p* < 0.05, ^@@@^ *p* < 0.005 as indicated. D) SEM images of blood cells on gauze, 2D, 3D, 0.5% RD‐3D, 1% RD‐3D, and 5% RD‐3D. Scale bar: 5 µm. E,F) APTT and PT evaluation of gauze, 2D, 3D, 0.5% RD‐3D, 1% RD‐3D, and 5% RD‐3D, with CaCl_2_ as positive control. *p < 0.05, **p < 0.01, ***p < 0.005 versus Gauze; ^&&^ *p* < 0.01, ^&&&^ *p* < 0.005 versus 2D; ^###^ *p* < 0.005 versus 3D; ^@^ *p* < 0.05 as indicated. Bars represent mean ± SEM of three independent experiments.

Achievement of effective wound healing requires immediate cessation of bleeding after skin injury, known as hemostasis. To evaluate the hemostatic efficacy, a mouse liver dissection model was established. As shown in **Figure** **3**A, bleeding time and blood loss were significantly reduced in the 0.5% RD‐3D (36.25 ± 3.68 s, 15.50 ± 0.49 mg), 1% RD‐3D (25.50 ± 2.02 s, 10.13 ± 0.67 mg), and 5% RD‐3D (21.50 ± 1.19 s, 8.53 ± 1.01 mg) group compared to the gauze group (58.00 ± 2.74 s, 25.48 ± 0.93 mg). Interestingly, the 2D membrane even showed delayed hemostasis and increased blood loss (63.00 ± 3.49 s, 28.65 ± 1.96 mg) compared to gauze. Although the 3D group without RD had a slightly accelerated blood absorption (55.25 ± 2.96 s, 22.55 ± 1.16 mg), there was no significant difference in bleeding time and blood loss in relative to gauze (Figure 3B,C). Taken together, these results suggest that RD‐3D dressings have excellent hemostatic activities in vivo.

In addition, during hemostasis, platelet activation and red blood cell (RBC) aggregation initiate the coagulation cascade, followed by fibrinogen conversion to fibrin as the final step. To gain insight into the hemostatic mechanisms of RD‐3D, we used SEM to examine platelet morphology and RBC surface adhesion. SEM images revealed that the number of RBC adhering or accumulating on the surface of the 3D scaffold was higher than on gauze and 2D membrane. Moreover, extensive platelet aggregation and activation, shown as protruding active pseudopodia, and fibrin formation were detected with increasing RD loaded on the 3D fibers, especially with 5% RD‐3D (Figure 3D). This observation suggested that RD‐3D scaffolds were not toxic to blood cells and exerted their coagulation capacity by enhancing platelet activation and aggregation. To further understand the involvement of extrinsic and intrinsic coagulation pathways in RD‐3D‐mediated hemostasis, we performed prothrombin time (PT) and activated partial thromboplastin time (APTT) assays independently at 37 °C. As shown in Figure 3E, there were weak effects of RD‐3D on APTT, indicating that RD‐3D had no significant impact on the intrinsic coagulation system. However, the RD‐3D groups significantly reduced PT in a dose‐dependent manner, suggesting that RD‐3D could exert anticoagulant activity through the extrinsic pathway (Figure 3F). In conclusion, RD‐3D fibrous dressings may help promote hemostasis by activating platelets to form blood clots in a bleeding wound scenario, where the extrinsic pathway is predominantly involved.

### 2.6 RD‐3D Fibrous Dressing as an Antibacterial and Inflammatory Regulator

**Figure 4:**
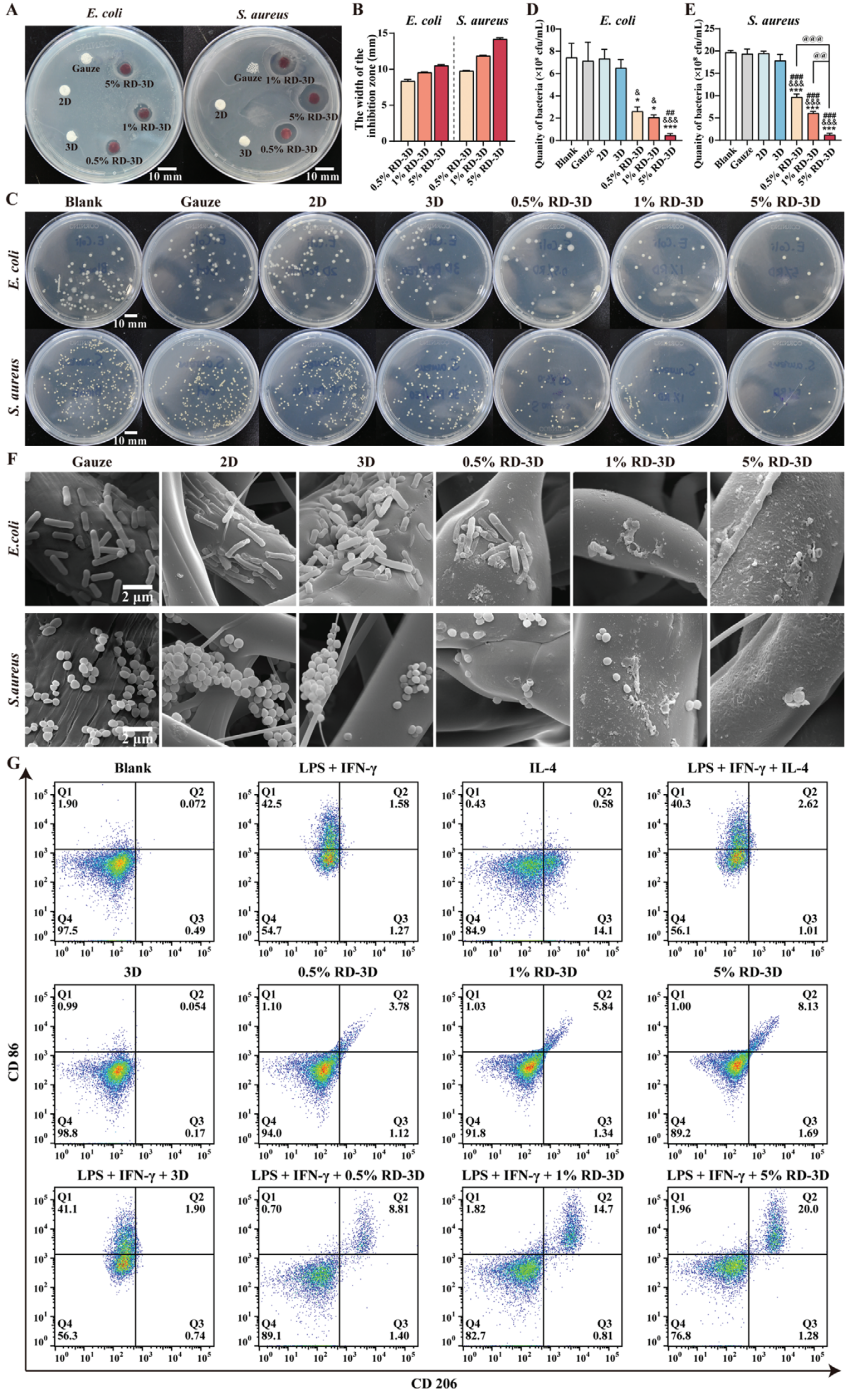
RD‐3D fibrous dressing as an antibacterial and inflammatory regulator. A) Agar plates showing inhibition zones of Gauze, 2D, 3D, 0.5% RD‐3D, 1% RD‐3D, and 5% RD‐3D against *E. coli* (left) and *S. aureus* (right), respectively. Scale bar: 10 mm. B) Quantification of inhibition zone widths in response to different dressings against *E. coli* and *S. aureus*. C) Images of colonies after co‐incubation of dressings with *E. coil* (upper panels) or *S. aureus* (lower panels) for 24 h, respectively. Scale bar: 10 mm. D,E) Quantification of bacterial colonies from (C) after 24 h co‐incubation of dressings with *E. coil* and *S. aureus*, respectively. *p < 0.05, ***p < 0.005 versus Gauze; ^&^ *p* < 0.05, ^&&&^ *p* < 0.005 versus 2D; ^##^ *p* < 0.01, ^###^ *p* < 0.005 versus 3D; ^@@^ *p* < 0.01, ^@@@^ *p* < 0.005 as indicated. F) SEM images of bacterial biofilms on dressing surfaces after 24 h co‐incubation in the bacterial suspension of *E. coli* (upper panels) or *S. aureus* (lower panels). Scale bar: 2 µm. G) Macrophage polarization of RAW 264.7 in response to LPS, IFN‐γ, 3D and RD‐3D fibrous dressing soaking solutions for 48 h, as detected by flow cytometry analysis using anti‐CD86‐PE and anti‐CD206‐Alexa Fluor 647 staining. Q1: CD86^+^/CD206^−^, M1 macrophages; Q2: CD86^+^/CD206^+^, M2b macrophages; Q3: CD86^−^/CD206^+^, M2 macrophages except for M2b; Q4: CD86^−^/CD206^−^, M0 macrophages. Bars represent mean ± SEM of three independent experiments.

Wound infections caused by bacterial pathogens, such as *Escherichia coli* (*E. coli*), *Staphylococcus aureus* (*S. aureus*), and *Pseudomonas aeruginosa* (*P. aeruginosa*), can lead to serious health complications, including delayed wound healing, severe pain, local or systemic inflammation, and even life‐threatening conditions.^[^ ^43^, ^44^, ^45^, ^46^ ^]^ Therefore, it is imperative to evaluate the antibacterial properties of biomaterials. Here, we investigated the potential antimicrobial activities of different types of dressings using *E. coli* and *S. aureus*, and the zone of inhibition (ZOI) method, which qualitatively measures the diffusivity of samples on culture plates. As shown in **Figure** **4**A,B, no ZOI was observed in the 6 mm in diameter gauze, 2D membrane and 3D scaffold groups for either *E. coli* or *S. aureus*. However, 0.5%, 1%, and 5% RD‐3D showed a strong effect against *E. coli*, with a ZOI radius of 8.36 ± 0.23, 9.56 ± 0.09, and 10.52 ± 0.12 mm, respectively. A similar phenomenon was observed for *S. aureus*, with the ZOI radius of 0.5%, 1%, and 5% RD‐3D being 9.77 ± 0.08, 11.86 ± 0.11, and 14.18 ± 0.18 mm, respectively, confirming the antibacterial activity of RD‐3D.

Previous studies have demonstrated that the antibacterial activity of RD could be attributed to the antioxidant property of RD and its interference with biomolecule synthesis and metabolic pathways.^[^ ^17^, ^26^, ^27^, ^47^ ^]^ To further validate the antibacterial ability of the RD‐3D dressings, we carried out a co‐culture setting with an aqueous environment, which allowed the dressings to come into full contact with bacteria in solutions. After the addition of gauze, 2D, 3D, and RD‐3D materials to *E. coli* or *S. aureus* solutions, the supernatants of the co‐cultures were applied to agar plates (Figure 4C). By quantifying the number of *E. coli* or *S. aureus* on the plates (Figure 4D,E), significant antibacterial effects were observed in the RD‐3D groups, which gradually increased with increasing RD load. Moreover, visualization of the morphology of *E. coli* and *S. aureus* on the impregnated fibers by SEM showed that the bacteria remained intact on the surface of the gauze, 2D and 3D scaffolds (Figure 4F). However, bacterial debris and altered bacterial membranes were observed in the fibers of the RD‐3D groups, and few unaffected bacteria remained on 1% RD‐3D and 5% RD‐3D fibers, particularly in the 5% RD‐3D condition, suggesting that RD‐containing scaffolds can directly disrupt the integrity of bacteria, thereby penetrating their membranes and exerting bactericidal effects. Taken together, these results demonstrate that RD‐3D is an excellent antibacterial dressing.

Given that antimicrobial responses are highly associated with the activation of M1 macrophages, an impaired transition of local macrophages from pro‐inflammatory M1 to anti‐inflammatory M2 phenotypes may result in nonhealing chronic wounds, leading to abnormalities such as decreased cell proliferation, angiogenesis, and collagen deposition in wound closure.^[^ ^48^ ^]^ To understand the role of RD‐3D in macrophage activation, we used RAW 264.7 macrophages to assess the effects RD‐3D on M1 or M2 polarization, for which CD86 and CD206 are typical biomarkers. As shown in Figure 4G, classical M1 macrophages are primarily activated by lipopolysaccharide/interferon‐γ (LPS/IFN‐γ), with a higher proportion of CD86^+^/CD206^−^ (Q1, 42.5%) compared to M2 CD86^−^/CD206^+^ macrophages (Q3, 1.27%). In contrast, in interleukin‐4 (IL‐4)‐induced M2 phenotypes, most commonly referred to as M2a, the percentage of CD86^−^/CD206^+^ macrophages was 14.1% compared to M1 CD86^+^/CD206^−^ at 0.43%. Although macrophages were still in a quiescent, nonactivated (M0) state in the presence of 3D fibers, the proportion of a specific subset of CD86^+^/CD206^+^ macrophages characterized as M2b, was shown when 3D was incorporated with RD (Q2, 3.78% in 0.5% RD‐3D, 5.84% in 1% RD‐3D, and 8.13% in 5% RD‐3D). Intriguingly, although RAW 264.7 cells were not affected by either M2‐triggering IL‐4 or 3D fibers in the presence of LPS/IFN‐γ, RD‐3D further polarized macrophages towards the M2b subtype in a dose‐dependent manner. The proportion of M2b increased from 8.81% in 0.5% RD‐3D to 14.7% in 1% RD‐3D and 20.0% in 5% RD‐3D, compared to the corresponding RD‐3D in the absence of LPS/IFN‐γ (3.78%, 5.84%, and 8.13%, respectively). Since M2b macrophages expressing both CD86 and CD206 can function as pro‐ and anti‐inflammatory cells that are essential for maintaining the balance of pro‐ and anti‐inflammatory factors in wounds,^[^ ^49^, ^50^, ^51^ ^]^ RD may contribute to the induction of the M2b phenotype from either M0 or M1.

### 2.7 Improved Pressure Ulcer Healing with RD‐3D Dressing

**Figure 5:**
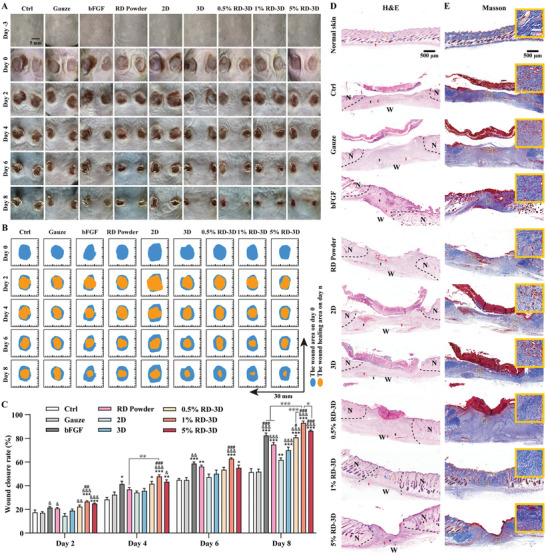
Improved pressure ulcer healing with RD‐3D dressing. A) Images of pressure ulcers after treatment with Gauze, recombinant bovine bFGF, RD powder, 2D, 3D, RD‐3D fibrous dressings on day −3, 0, 2, 4, 6, and 8. Scale bar: 5 mm. B) Traces of wound closure on day 0, 2, 4, 6, and 8. C) Quantification of wound size, *n* = 3. *p < 0.05, **p < 0.01, ***p < 0.005 versus Gauze; ^&^ *p* < 0.05, ^&&^ *p* < 0.01, ^&&&^ *p* < 0.005 versus 2D; ^#^ *p* < 0.05, ^##^ *p* < 0.01, ^###^ *p* < 0.005 versus 3D; ^@^ *p* < 0.05, ^@@^ *p* < 0.01, ^@@@^ *p* < 0.005 as indicated. D) H&E staining images of wound sections on day 8. Black dashed lines: boundaries between wounds and normal skin; N: normal skin; W: wounds. Arrow in red: blood vessels; in blue: hair follicles; in yellow: sebaceous glands; in black: inflammatory cells. Scale bar: 500 µm. E) Masson's trichrome staining images of wound sections on day 8. Images with yellow solid border are magnifications of areas with dashed frame. Scale bar: 500 µm. Data are presented as mean ± SEM.

As the preferred structure and potent bioactivity in hemostasis and inflammatory regulation suggest RD‐3D as a suitable dressing for wound healing, we evaluated the efficacy of RD‐3D fibrous dressings on pressure ulcer models established on the back of shaved mice and measured wound size every 2 d for a continuous period of 8 d (**Figure** **5**A–C). Throughout the treatment period, wounds exposed to 3D dressings showed slightly more efficient healing than those exposed to 2D membranes. Moreover, unlike 3D dressings, which showed significantly faster wound closure from day 8, RD power and 3D‐RD showed efficacy as early as day 2, particularly for RD‐3D at 1% and 5%. In addition, there was less scab formation in the healed wounds after application of RD‐3D (Figure 5A). Pathologically, hematoxylin and eosin (H&E) staining revealed that the untreated control (Ctrl) had the detachment of scabs and a granulation tissue formation accompanied by large numbers of inflammatory cells (Figure 5D). Similarly, wounds exposed to gauze showed a visible structure of the pressure ulcer and newborn granulation tissue. Although recombinant bovine basic fibroblast growth factor (bFGF) gel exposure had resulted in gradual remodeling of the wounds into normal skin, a thicker epidermis and thinner dermis were noted. In the 3D fibers without RD, a layer of new keratin‐forming cells was observed along with a large number of inflammatory cells. However, the RD powder without 3D fibers showed recovered wounds with a clear boundary between the subepidermal granulation tissue and the surrounding newborn dermis. Thus, RD‐3D treatment resulted in normal epidermal and dermal structures, accompanied by subepidermal neoplastic hair follicles with sebaceous glands. Using Masson's trichrome staining (Figure 5E), only RD‐3D treatment restored a dense and organized pattern of collagen fibrils, indicating favorable wound healing. Meanwhile, H&E staining for liver, kidney, and spleen showed no injury from topical application of RD‐3D (Figure S3, Supporting Information), suggesting that RD‐3D is a promising option for cutaneous wounds such as pressure ulcers.

### 2.8 Accelerated Healing of Infected Wounds with RD‐3D Dressing

**Figure 6:**
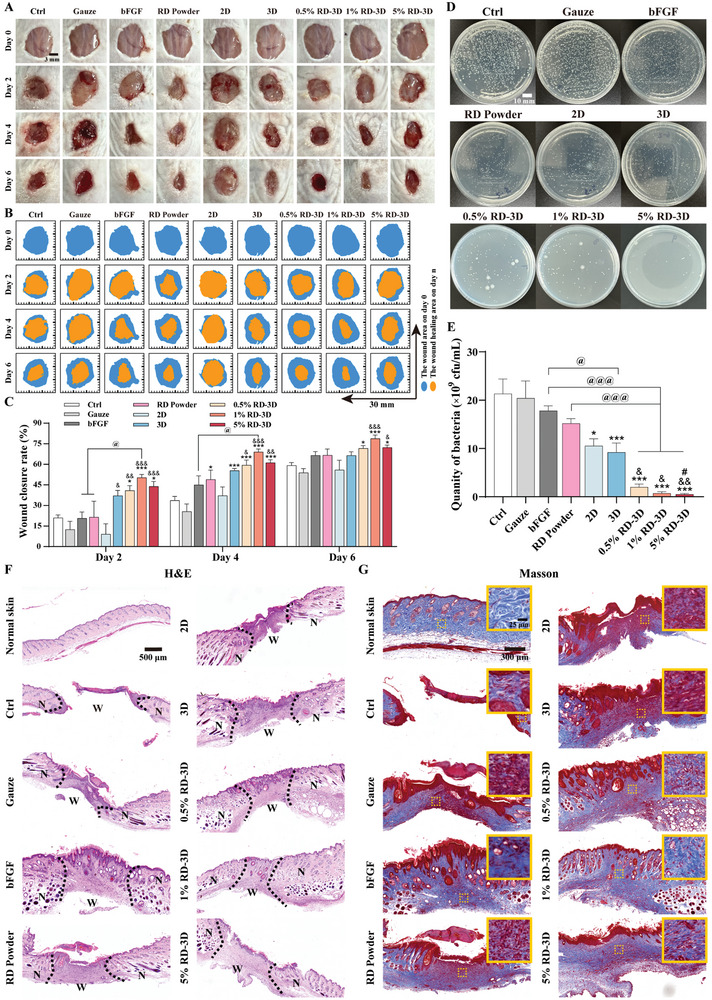
Accelerated healing of infected wounds with RD‐3D dressing. A) Images of *S. aureus‐*infected wounds treated with Gauze, recombinant bovine bFGF, RD powder, 2D, 3D, 0.5% RD‐3D, 1% RD‐3D, or 5% RD‐3D, on day 0, 2, 4, and 6, respectively. Scale bar: 3 mm. B) Traces of wound closure on day 0, 2, 4, and 6. C) Quantification of wound size, *n* = 6. *p < 0.05, ***p < 0.005 versus Gauze; ^&^ *p* < 0.05, ^&&^ *p* < 0.01, ^&&&^ *p* < 0.005 versus 2D; ^@^ *p* < 0.05 as indicated. D) Images of bacterial colonies derived from LB‐cultured dressings at the end of the last administration, representing wound infection on day 6. Scale bar: 10 mm. E) Quantification of bacterial colonies from (D), *n* = 6. *p < 0.05, ***p < 0.005 versus Gauze; ^&^ *p* < 0.05, ^&&^ *p* < 0.01 versus 2D; ^@^ *p* < 0.05, ^@@@^ *p* < 0.005 as indicated. F) H&E staining images of wound sections on day 6. Black dashed lines: boundaries between wounds and normal skin; yellow dashed lines: wound area; N: normal skin; W: wounds. Scale bar: 500 µm. G) Masson's trichrome staining images of wound sections on day 6. Images with yellow solid border are magnifications of areas with dashed frame. Scale bar: 300 µm. Data are presented as mean ± SEM.

Based on the excellent antibacterial performance of RD‐3D (Figure 4), a more complex chronic wound model of *S. aureus* infection was established and continuously monitored for 6 and 12 d (**Figures** **6**A–C and S4, Supporting Information). In contrast to the pressure ulcer models, infected wounds treated with either 3D fibers, or 0.5%, 1%, and 5% RD‐3D dressings had dramatic impacts on early healing stage (day 2 vs day 0), showing a reduction in wound size of 37.06 ± 3.92%, 40.89 ± 3.50%, 50.34 ± 2.30%, and 43.92 ± 3.59%, respectively (Figure 6C). The benefits of RD in 3D fibers became more apparent on day 4 and day 6, in terms of better closure of infected wounds and absence of redness, swelling and suppuration, particularly with 1% RD‐3D (Figure 6A,B). During this process, we had noticed that both gauze and 2D fibers were not suitable for healing skin wounds due to secondary damage during dressing changes, confirming the advantages of the coiled and porous structure of 3D dressings. Moreover, the dressings were collected after treatment on day 2, day 4 (Figure S5, Supporting Information), and day 6 (Figure 6D,E) and evaluated for their antibacterial properties. In contrast to the significant bacterial proliferation in the control, gauze, recombinant bovine bFGF, 2D, and 3D groups (Figure 6D,E and Figure S5, Supporting Information), the RD‐3D dressings exhibited excellent antibacterial activity, with more than 90% bactericidal efficacy in 5% RD‐3D on day 6. Pathological examination further confirmed that all treatments, with the exception of the control which had a prominent scab, resulted in new epidermis and granulation tissue by day 6 (Figure 6F). Comparing RD‐3D with the positive control bFGF, which has no antibacterial effect, at day 12 (Figure S4, Supporting Information), both resulted in visible hair follicles and glands, but a thicker epidermis was observed in the bFGF group (Figure S4D, Supporting Information). A similar trend was observed for collagen deposition at the wound site on day 6 and day 12 (Figure 6G and Figure S4E, Supporting Information), where collagen was found to be structurally intact and wavy, with a higher density and homogeneous distribution in RD‐3D, typically in 1% RD‐3D. In addition, pathological evaluations of the liver, kidney and spleen confirmed the safety of the RD‐3D dressings during the 6‐d (Figure S6, Supporting Information) and 12‐d process (Figure S7, Supporting Information). Collectively, these data support both the 3D topography and the effectively loaded RD that actively promotes wound healing, and RD‐3D is an appropriate healthcare dressing for infected wounds.

### 2.9 Contribution of key RD Components to Wound Healing In Vitro

**Figure 7:**
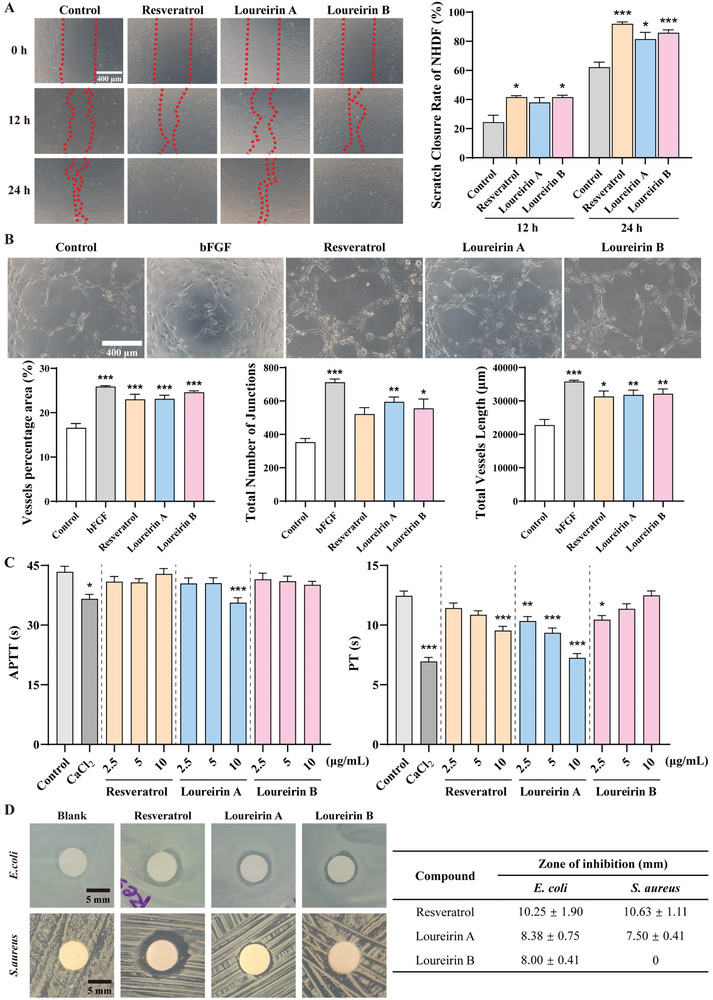
Contribution of key RD components to wound healing in vitro. A) Images of cell scratch (left) and scratch closure rate (right) of NHDF treated with resveratrol (Res), loureirin A (LA), and loureirin B (LB) at 0, 12, and 24 h. The red dashed lines are the cell migration boundary. Scale bar: 400 µm. *p < 0.05, ***p < 0.005 versus Control. B) Upper panels are images of tube formation of HUVEC treated with recombinant human bFGF, Res, LA, or LB for 6 h, with bFGF as a positive control. Scale bar: 400 µm. Bottom panels show quantification of percentage vessel area (left), total number of junctions (middle), and total vessel length (right) of images. *p < 0.05, **p < 0.01, ***p < 0.005 versus Control. C) Evaluation of APTT and PT exposed to Res, LA, and LB, with CaCl_2_ as a positive control. *p < 0.05, **p < 0.01, ***p < 0.005 versus Control. D) Images (left) and corresponding quantifications (right) of inhibition zones of filter paper, Res, LA, and LB against *E. coli* (upper panels) and *S. aureus* (lower panels), respectively. Scale bar: 5 mm. Data are presented as mean ± SEM of three independent experiments.

Resveratrol, loureirin A, and loureirin B are the major components of RD that could be efficiently released from RD‐3D (Figure 1H). Following the cytotoxicity of resveratrol, loureirin A, and loureirin B in HaCat, NHDF, and HUVEC cells in vitro (Figure S8, Supporting Information), we sought to determine the individual contribution of these components to wound healing. Firstly, all three compounds were able to induce cell proliferation after 24 h incubation, suggesting a general role for these compounds in promoting granulation, angiogenesis, and re‐epithelization. As re‐epithelialization generally requires the migration of cells such as fibroblasts at the leading edge of the wound to promote healing, we next evaluated their effects on NHDF migration using the scratch wound healing assay. As shown in **Figure** **7**A, all three components promoted the scratch wound healing of NHDF faster than the control, especially by resveratrol after 24 h of treatment. Secondly, we used 3D Matrigel assays to investigate the angiogenic properties of the three components by measuring the formation of tubular structures. As shown in Figure 7B, recombinant human bFGF as a positive control induced a massive, elongated tube‐like structure. Consistent with recombinant human bFGF, a general induction of tube formation, in terms of vessels, total connections, and total vessel length, was obtained in all conditions exposed to the bioactive components of RD, among which loureirin A showed the greatest potency. Thirdly, we tested the hemostasis induced by RD components. As shown in Figure 7C, only treatment with loureirin A at 10 µg mL^−1^ showed a significant reduction in APTT, and both resveratrol and loureirin A were found to reduce PT in a dose‐dependent manner. These results suggest that loureirin A may be involved in hemostasis through both intrinsic and extrinsic pathways. Finally, in terms of antimicrobial properties, resveratrol again showed promising values in protecting against infection by both *E. coli* and *S. aureus* (Figure 7D), consistent with previous studies of its broad‐spectrum antimicrobial activity.^[^ ^26^ ^]^ In sum, these results suggest that the bioactive components of RD have distinct wound‐healing properties that collectively benefit different stages of wound healing.

## 3 Conclusions

In summary, a 3D fibrous resin of botanical RD has been electrospun for chronic wound healing. The developed RD‐3D dressings have a coiled structure with nanopores to provide gas permeability, exudate absorption, a moist environment, and to prevent secondary injury during dressing changes. Moreover, the application of electrospinning to RD overcomes the limitations of conventional RD powders or ointments, which are unfavorable for wound healing due to the lack of oxygen exchange and moist environment necessary for the wound healing process. In addition, the integration of RD has resulted in accelerated hemostasis, enhanced bactericidal activity, increased cell proliferation and migration, and a balanced pro‐and anti‐inflammatory environment to promote rapid repair of both pressure ulcers and infected wounds. As the main components of RD, resveratrol was confirmed to contribute to bactericidal effects and cell migration, loureirin A was involved in vascularization and hemostasis, and loureirin B together with resveratrol and loureirin A promoted wound closure. Overall, the electrospun bioactive dressing is a promising candidate for chronic wound healing and may improve upon conventional 2D dressings and topical RD formulations in future clinical practice.

## 4 Experimental Section

### Materials

RD was purchased from Xishuangbanna Pharmaceutical Co., Ltd. (220114, Yunnan, China). The reference standards of resveratrol (≥98% purity, HPLC), loureirin A (≥98% purity, HPLC), and loureirin B (≥98% purity, HPLC) were purchased from Yuanye Bio‐Technology (Shanghai, China). PCL of 80 kDa (in number Mn), PEO (Mv = 300 kDa), sodium dodecyl sulfate (SDS), and dimethyl sulfoxide (DMSO) were obtained from Sigma‐Aldrich (St. Louis, MO, USA). Trichloromethane was supplied from China National Pharmaceutical Group Corporation (Beijing, China). Anhydrous ethanol (AR grade) was purchased from Beijing Chemical Works (Beijing, China). Acetic acid was supplied from Aladdin Biochemical Technology (Shanghai, China), and acetonitrile (HPLC grade) was purchased from Thermo Fisher Scientific (Wilmington, MA, USA).

### The Fabrication of 3D Fibrous Dressings

15 wt% PCL/PEO was weighed and dissolved in trichloromethane following the ratios 100:0, 95:5, 90:10, and 80:20 (w/w), respectively. The solution was stirred for 12 h at room temperature (RT). The 5 mL syringe filled with different ratios of PCL/PEO solution was placed horizontally, and the fibers were electrospun using an electrospinning machine (ET‐2535DC, Ucalery, Beijing, China) under the following conditions: voltage of 8.5–12 kV, needle gauge of 20, flow rate of 0.12–0.15 mm min^−1^, distance from needle tip to collector between 10 and 11 cm, temperature of 30 ± 5 °C, and humidity in the range of 15%–30%. Detailed information on the electrospinning parameters is given in Table S1 (Supporting Information). The spinning was performed on collectors including 1) anhydrous ethanol, 2) anhydrous ethanol with RD, and 3) a flat plate. After spinning, the obtained fibrous scaffolds were washed with ddH_2_O and kept overnight at −20 °C for solidification. The samples were then freeze‐dried for 24 h by a freeze‐drying system (LGJ‐10C, Foring Technology, Beijing, China).

### Scanning Electron Microscopy (SEM)

The fibrous dressings were sputter‐coated with a thin layer of gold before examination. The microstructures of the samples were observed by using a field emission scanning electron microscopy (Phenom Pharos G2, Eindhoven, Netherlands) at an accelerating voltage of 10 kV. The diameters of the resulting fibers were measured from randomly selected fibers on SEM image. A total of 150 fibers were measured from each sample, and the average diameter was calculated using image analysis software (Image J, NIH, USA).

The interaction between samples and blood cells was investigated by SEM. Briefly, the blood (50 µL) was dropped onto the sample surface. After the samples were placed in a 37 °C incubator for 20 min and rinsed with PBS, the blood cells on the samples were fixed with 2.5% glutaraldehyde (Macklin, Shanghai, China) for 1 h and further dehydration with a series of ethanol with different concentrations (30%, 50%, 70%, 80%, 90%, 95%, and 100%) for SEM observation.

### Water Contact Angle

The contact angles of the PCL/PEO membranes were determined by Theta Flow (Biolin Scientific, Espoo, Finland) with the sessile drop method. Briefly, a droplet volume of 3 µL ddH_2_O and a recording time of 1 s were used to measure the water contact angle. The average contact angle was calculated from three independent experiments.

### Swelling Index

The electrospun fibrous dressings were weighed and placed in a 2 mL solution with PBS:ethanol (v/v, 1:1) containing 0.1% SDS. The samples were stirred at 37 °C by shaking at 200 rpm for 24 h. Excess moisture was removed by blotting with filter paper, and the weight was recorded. The swelling index was calculated according to the following formula [Equation (1)]

(1) $$\mathrm{Swelling}\;\mathrm{index}\;=\frac{W2-W1}{W1}\;\times 100\%$$

W2 is the mass after absorption and W1 is the mass before absorption.

### Porosity

The total porosity of scaffolds can be determined by gravimetric method using the apparent density of the scaffold and bulk density of the material according to the following equation [Equation (2)]^[^ ^32^ ^]^

(2) $$\mathrm{Porosity}\;=\left(1-\frac{\rho }{\mathrm{bulk}\;\mathrm{density}}\right)\;\times 100\%$$

where apparent density (ρ) of scaffolds is defined as the mass divided by the volume of porous scaffolds according to the equation of ρ  =  m/V.

### Mechanical Properties

The mechanical properties of the electrospun samples were measured using the universal testing machine (SHIMADZU, Kyoto, Japan) with a test load of 10 N and a tensile speed of 10 mm min^−1^. Rectangular sections of the samples (10 mm × 15 mm) with a thickness of 0.25 mm were mounted (*n* = 3 for each sample). Tensile strength, elongation at break, and Young's modulus were determined from the stress–strain curves generated.

### Reversed‐Phase High‐Performance Liquid Chromatography

The levels of resveratrol, loureirin A, and loureirin B in the samples were determined by Vanquish Core HPLC system (Thermo Fisher Scientific, Bremen, Germany). Chromatography was performed using a C18 column as stationary phase (Agilent, Stable Bond 300, 250 mm × 4.6 mm, 5 µm). The mobile phase consisted of A (0.2% acetic acid) and B (acetonitrile) and was delivered using a gradient elution from 20% B to 45% B within 55 min at a flow rate of 1 mL min^−1^. Detection was performed at a wavelength of 270 nm, and the concentration of each compound was determined using a calibration curve.

Standards for resveratrol, loureirin A, and loureirin B were prepared for calibration curves. Briefly, 1 mg mL^−1^ of each compound was dissolved in HPLC acetonitrile and subjected to serial dilutions. The solutions were then filtered through a 0.45 µm membrane filter (Millipore, Massachusetts, USA) before injection into the RP‐HPLC system. The retention times of the standard references for resveratrol, loureirin A, and loureirin B were 16, 49, and 51 min, respectively. Calibration curves were generated with a linear relationship between peak area and concentration.

### Fourier Transform Infrared Spectroscopy

The chemical bonds of RD, 3D, and RD‐3D were characterized using an FTIR spectrometer (SHIMADZU, Kyoto, Japan) at RT in the attenuated total reflection (ATR) mode. The infrared spectra were recorded at 4000 to 400 cm^−1^.

### In Vitro Drug Release Study

Release study on resveratrol, loureirin A, and loureirin B from 5% RD‐3D (400 mg) was carried out at a temperature of 37 °C in an 8 mL solution containing 50% ethanol and 1% (w/v) SDS at a stirring speed of 200 rpm. 1 mL solution was collected at frequent intervals (15, 30, and 45 min, and 1, 2, 3, 6, 9, 12, 24, 36, and 48 h), after each collection, 1 mL of fresh solution was added to maintain a total volume of 8 mL. RP‐HPLC was used to analyze the amount of resveratrol, loureirin A, and loureirin B that was analyzed.

### DPPH Radical Scavenging Experiment

5 mg of fibrous dressing was weighed and added to the 10^−4^ m DPPH ethanol solution. The concentration of Resina draconis was determined based on the incorporation efficiency of 0.5%, 1%, and 5% RD in the coagulation bath during RD‐3D electrospinning, corresponding to 500, 1000, and 2000 µg mL^−1^ loaded RD, respectively. The absorbance at 517 nm was measured after 10 min of reaction at RT in the dark DPPH scavenging activity was calculated according to the following formula [Equation (3)]

(3) $$\mathrm{DPPH}\;\mathrm{scavenging}\;\mathrm{activity}\;=\left(1-\frac{\mathrm{Ai}-\mathrm{Aj}}{\mathrm{Ac}}\right)\;\times 100\%$$

Ac is the blank value, Ai is the sample group, and Aj is the control group.

### Drug Loading Efficiency

RD‐3D was weighed into 5 mL of chloroform‐ethanol (v/v, 7:3), sonicated until the fibrous dressings were completely dissolved, then 7 mL ethanol was slowly added, followed by centrifugation at 4 °C and 4500 rpm for 15 min. After centrifugation, the supernatant was fixed with ethanol to 12 mL, which was dried in a vacuum at RT for 24 h. It was redissolved with ethanol until the liquid was completely evaporated. After centrifugation and volatilization of the supernatant, the dry matter was weighed to obtain the exact amount of RD from the scaffolds.

### Cell Culture

HaCat were obtained from FuHeng Cell Center (Shanghai, China) and grown in DMEM medium (Gibco) supplemented with 10% fetal bovine serum (FBS, Gibco) and 1% penicillin/streptomycin (P/S, Gibco). NHDF were purchased from BeNa Culture Collection (BNCC, Beijing, China) and expanded in DMEM medium supplemented with 10% FBS and 1% P/S. HUVEC were obtained from ScienCell Research Laboratories (San Diego, CA) and expanded in complete endothelial cell medium (ECM, ScienCell) supplemented with 10% FBS (ScienCell), 1% ECGS (ScienCell), and 1% P/S (ScienCell). RAW 264.7 was purchased from Procell Life Science & Technology (Procell, Wuhan, Hubei, China) and expanded in DMEM medium supplemented with 10% FBS and 1% P/S. All cells were incubated at 37 °C in a humidified atmosphere with 5% CO_2_. Cells were enzymatically treated with Trypsin‐EDTA (0.25%, Gibco) for passaging every 5–7 d.

### Preparation of Fibrous Dressing Soaking Solutions

Fibrous dressings were sterilized by UV irradiation before sealing. DMEM medium containing 10% FBS and 1% P/S was used as the extraction medium, which was added at 3 mg mL^−1^, and then placed in the incubator at 37 °C with 5% CO_2_ for 12 h and stored at 4 °C.

### Cell Viability and Proliferation

Live/dead staining was performed to visualize the number of viable and nonviable HaCat, NHDF, and HUVEC cells. After 24 h cultivation in the soaking solutions of fibrous dressings, samples were stained with PBS containing 2 × 10^−3^ m Calcein‐AM (Invitrogen, Carlsbad, CA) and 4 × 10^−3^ m propidium iodide (PI, Sigma) for 10 min at 37 °C. Calcein‐AM reacts with the intracellular esterase of live cells to emit green fluorescence, while PI interacts with DNA of dead cells with red fluorescence. Digital images of viable (green) and dead (red) cells were visualized using an Olympus FV3000 confocal laser microscope (Tokyo, Japan).

Cell proliferation was assayed with CellTiter96Aqueous One Solution Cell Proliferation Assay Kit (Promega, Madison, WI). Briefly, cells were seeded and cultured in the medium supplemented with various compounds (resveratrol, loureirin A, and loureirin B) as indicated concentrations at a density of 5 × 10^4^ HaCat cells cm^−2^, 3 × 10^4^ NHDF cells cm^−2^, or 2 × 10^4^ HUVEC cells cm^−2^ for 24 h. Then, the cell culture medium was removed, MTS reagent was added to each well and incubated for 3 h. The same volumes of culture medium and MTS‐reagent without cells were also incubated as the background. 100 µL of the solution was transferred into a 96‐well plate, and the absorbance at 490 nm was measured for each well. Alternatively, cells were seeded at the same density and cultured in the soaking solutions of fibrous dressings. The cell proliferation was examined on day 1, 3, and 5 after incubation.

### Cell Migration Experiment

HaCat or NHDF (3.5 × 10^4^ cells/well) were seeded onto two‐well culture inserts (Ibidi GmbH, Gräfelfing, Germany) and incubated at 37 °C to allow full cell growth and formation of a single cell layer. The inserts were removed, and the cells were rinsed with PBS. 1 mL medium was added to each well with pictures taken at regular intervals (0, 12, and 24 h). The scratch closure rate was calculated using the following formula [Equation (4)]

(4) $$\mathrm{Scratch}\;\mathrm{closure}\;\mathrm{rate}\;=\frac{A_{0}-A_{n}}{A_{0}}\;\times 100\%$$

*A*_0_ is scratch area at the beginning, and *A_n_* is scratch area at n hour.

The scratch data were used for relative statistics of area with Image J software.

### Tube Formation Experiment

10 µL Matrigel (R&D Systems, MN, USA) was placed in each well of angiogenesis µ‐slides (Ibidi GmbH, Gräfelfing, Germany) for 1 h at 37 °C. Then, HUVEC at a density of 1 × 10^4^ cells/well was added with 35 ng mL^−1^ recombinant human bFGF (R&D) or compounds (resveratrol, loureirin A, and loureirin B). After incubating for 8 h at 37 °C in a humidified atmosphere with 5% CO_2_, the pictures were taken and analyzed using AngioTool (NIH, USA).

### Macrophage Polarization Experiment

For pro‐inflammation macrophage activation, RAW 264.7 was treated with 200 ng mL^−1^ LPS (BIORIGIN, Beijing, China) and 2.5 ng mL^−1^ IFN‐γ (novoprotein, Jiangsu, China) for 48 h. Conversely, to activate anti‐inflammation macrophage, RAW 264.7 was treated with 10 ng mL^−1^ IL‐4 (Abcam, Cambridge, UK) for 48 h. To evaluate the effect of experimental samples on the polarization of macrophages, RAW 264.7 cells were cultured with IL‐4, 3D and RD‐3D fibrous dressings (0.5%, 1%, and 5% RD‐3D) soaking solutions with or without LPS/IFN‐γ for 48 h, respectively. The cells were then collected and fixed with 4% paraformaldehyde (Macklin), and permeabilized with 0.1% triton X‐100 (Sigma) in PBS. After this, extracellular staining with anti‐CD86‐PE (BD, CA, USA) and intracellular staining with anti‐CD206‐Alexa Fluor 647 (BD) were performed. Following a wash, the cells were analyzed using a flow cytometer (FACSCelesta, BD), and at least 10,000 events were collected for each sample. The data were analyzed with FlowJo software (V.10.4, BD).

### Hemocompatibility

5 mg mL^−1^ of gauze or fibrous dressings were incubated with diluted whole blood of SD rats (5% PBS, v/v) at 37 °C for 1 h. The above mixture was centrifuged at 1000× *g* for 10 min to collect the supernatant, and the absorbance at 540 nm was determined. PBS (10% PBS, v/v) and double‐distilled water (5% ddH_2_O, v/v) were used as negative and positive controls. The hemolysis rate was calculated according to the following formula [Equation (5)]

(5) $$\mathrm{Hemolysis}\;\mathrm{rate}\;=\left(\frac{\mathrm{ODsample}-\mathrm{ODnegative}}{\mathrm{ODpositive}-\mathrm{ODnegative}}\right)\;\times 100\%$$

### APTT and PT

APTT and PT were determined by APTT test kit (Yuanye, Shanghai, China) and PT test kit (Leagene, Beijing, China), respectively. By detecting APTT and PT, it was judged whether fibrous dressings activate the intrinsic or extrinsic coagulation pathway. Platelet‐poor plasma (PPP) was obtained by centrifuging anticoagulated whole blood at 2000× *g* for 10 min at 4 °C. The fibrous dressings (5 mg mL^−1^), Resina draconis, resveratrol, loureirin A, and loureirin B were mixed with PPP and incubated at 37 °C for 30 min. The sample‐removed plasma (100 µL) was then mixed with APTT reagent (ellagic acid, 100 µL) and incubated at 37 °C for 5 min. Subsequently, the preheated CaCl_2_ solution (100 µL, 25 × 10^−3^ m) was added to the above mixture, and the time was measured immediately, and the plasma coagulation time was recorded as APTT. The plasma (70 µL) to be tested according to the above procedure was incubated at 37 °C for 3 min. After adding the preheated PT reagent (140 µL) to the plasma, the time was measured immediately, and the plasma coagulation time was recorded as PT.

### Bacterial Culture

*E. coli* and *S. aureus* (testobio, Ningbo, Zhejiang, China) were cultured in Luria‐Bertani (LB) broth on a shaker at 150 rpm at 37 °C for overnight. The LB broth medium is composed of 1% (w/v) tryptone (Oxoid, Basingstoke, UK), 1% (w/v) NaCl (Sigma), and 0.5% (w/v) yeast extract (Oxoid) in ddH_2_O, then adjusted at pH 7.4 with NaOH (Aladdin). The broth was autoclaved at 121 °C for 30 min and stored at 4 °C. LB agar plate was prepared by LB broth with 1.5% (w/v) agar (Biowest, Nuaille, France) in petri dishes and stored at 4 °C before use.

### Co‐culture Investigation

The gauze and fibrous dressings samples were co‐cultured with *E. coli* and *S. aureus* (testobio, Ningbo, Zhejiang, China) suspensions (1 × 10^6^ CFU mL^−1^) at 37 °C for 24 h. Then the two bacterial solutions were diluted 1 × 10^6^ times and spread evenly in the nutrient LB agar plates. The number of colonies was recorded accordingly after incubation for 24 h. After co‐culturing, the samples were rinsed with PBS. The bacteria on the samples were then fixed with 2.5% glutaraldehyde for 1 h and further dehydration for SEM observation.

### Inhibition Zones Experiment

Gauze, fibrous dressings, and filter papers were punched into uniform circular discs with diameters of 6 mm using a hole puncher. The filter paper was soaked in an ethanol solution containing the compounds (resveratrol, loureirin A, and loureirin B), and left to dry in a ventilated place away from light. The discs were then placed under a UV lamp for 30 min to sterilize them. Sterile nutrient agar plates were prepared, and 0.1 mL of the bacterial suspensions (1 × 10^8^ CFU mL^−1^) was evenly spread onto each plate to obtain plates with bacteria. The sterilized materials were placed on the agar plates with bacteria and incubated inverted at 37 °C for 18 h. The inhibition zones were observed, and the width of the inhibition zones was recorded.

### In Vivo Wound Healing Experiment

C57BL/6J mice (male, 6–8 weeks, 20 ± 1 g) were used to evaluate the wound healing potential of dressings in vivo. The mice were given a standard diet and free water during a 12 h light‐dark cycle at a temperature of 22 ± 2 °C and relative humidity of 50 ± 10%. All animal studies were performed in accordance with the National Guidelines for Care of Laboratory Animals and approved by the Institutional Animal Care and Use Committee of the Institute of Basic Theory for Chinese Medicine, China Academy of Chinese Medical Sciences (Approval No. IBTCMCACMS21‐2305‐02).

The hemostatic abilities of samples were assessed by the hepatic hemorrhage model in mice. Twenty‐four C57BL/6J mice were randomly divided into six groups after adaptive feeding for 7 d. The mice were anesthetized with an intraperitoneal injection of 75 mg kg^−1^ Zoletil 50 (Virbac, Cedex, France) and 10 mg kg^−1^ Xylazine (Huamu Corp., Changchun, Jilin, China). After exposing the liver with surgical scissors, a wound (0.5 cm length, 0.1–0.2 cm depth) was created on the liver with a scalpel. After the wound naturally bled for 3 s, the outflowing blood was sucked with a weighed sterile gauze or 30 mg of fibrous dressings samples sterilized by ultraviolet radiation were immediately completely covered on the wound surface and then the bleeding was observed every 30 s. If no active bleeding was observed at the wound within 15 s, the hemostasis was considered successful and the hemostasis time and the amount of blood loss at the wound site were then recorded.

A study was conducted to evaluate the effectiveness of fibrous dressings in treating chronic wounds with impaired local blood circulation. A pressure ulcer model in mice (C57BL/6J) was created. Twenty‐seven C57BL/6J mice were randomly divided into nine groups before surgery. Specifically, the dorsal skin of mice was shaved and pinched by the paired magnets (8 mm in diameter, 1.5 mm in thickness) to cause ischemia for 12 h and then removed for 12 h to allow reperfusion. Three cycles of ischemia‐reperfusion (IR) were introduced to create this model. Dressings were then applied to the injured skin and changed every 2 d. The control group was treated with adhesive tape only. Various types of dressings, gels, and powders were applied topically to the wounds. These included gauze, recombinant bovine bFGF gel (ESSEX, Zhuhai, Guangdong), 2D membrane, 3D scaffolds, RD powder, and RD‐3D (0.5%, 1%, and 5%) dressings. Mice treated as positive controls received 6 mg bFGF gel (300 IU cm^−2^), according to the manufacturer's instructions and previous studies.^[^ ^33^ ^]^ In addition, tested mice received 6 mg RD powder, 30 mg 0.5% RD‐3D (3 mg RD), 30 mg 1% RD‐3D (6 mg RD), or 30 mg 5% RD‐3D (12 mg RD) for evaluation. Photographs were taken to record the wound's progress.

A more complex infected wound healing behavior was evaluated by covering different dressings onto the infectious skin wound of mice. Twenty‐seven C57BL/6J mice were randomly divided into nine groups before surgery. The mice were anesthetized by intraperitoneal injection, and then the dorsal region of the mice was shaved with a razor and cleaned with depilatory cream (Veet, Paris, France). A circular full‐thickness wound was generated on the dorsal area of each mouse, and 50 µL of 1 × 10^8^ CFU mL^−1^ *S. aureus* suspension was added to the wound. Dressings were then applied to the injured area and typically changed every 2 d. Photographs were taken to document the wound‐healing process. After changing the dressings, samples were collected and cultured in 1 mL of liquid medium at 37 °C for 24 h. The bacterial solution was then diluted 1 × 10^6^ times and spread evenly on nutrient agar plates. After incubation for 24 h, the number of colonies was recorded.

The wound closure rate was calculated according to the following formula [Equation (6)]

(6) $$\mathrm{Wound}\;\mathrm{closure}\;\mathrm{rate}\;=\left(\frac{S_{n}-S_{0}}{S_{0}}\right)\;\times 100\%$$

*S*_0_ is wound area at the beginning, and *S_n_* is wound area on day *n*.

The wound closure data were used for relative statistics of area with Image J software.

### Histological Staining

The wound tissues were removed and fixed with formaldehyde (4%) in PBS buffer for 2 d. Then the biopsies were embedded in paraffin and sectioned into 6‐µm slices using a microtome (Leica, Nussloch, Germany). After deparaffinized and rehydrated, the tissue sections were stained with H&E staining solution (Solarbio) and Masson's trichrome stain kit (Solarbio), according to the manufacturer's protocol. All images were taken using the ScanScope slide scanner (Aperio, CA, USA) with an image analysis system (Aperio V.12.4.3.5008).

### Statistical Analysis

Data are represented as mean value ± the standard error of the mean and were analyzed with the one‐way analysis of variance (ANOVA) with the Bonferroni post hoc test. Values are considered to be statistically significant at *p* < 0.05. Statistical analysis and figures were created using Prism 9 (GraphPad, San Diego, CA, USA).

## Conflict of Interest

The authors declare no conflict of interest.

## Author Contributions

R.X. and N.L. designed the processes and experiments. S.G., P.W., Y.S., C.C., J.G., and S.H. were involved in preparing samples and performing experiments. R.X., N.L., and S.G. analyzed and interpreted data. R.X. and N.L. supervised the overall research. R.X., N.L., S.G., and S.Y. contributed to the writing of the manuscript. All authors contributed and approved the submitted version.

## Supporting information

Supporting Information

## Data Availability

The data that support the findings of this study are available in the Supporting Information of this article.
