# Supplementary material for: Transformation of Natural Resin Resina Draconis to 3D Functionalized Fibrous Scaffolds for Efficient Chronic Wound Healing
Source: Adv Healthc Mater. 2024 Jun 25;13(30):2401105. doi: 10.1002/adhm.202401105 (PMC11616260; doi:10.1002/adhm.202401105)
Supplement: Supplementary file 1 — Supporting Information [file ADHM-13-0-s001.docx]

**Supporting Information**

**Transformation of Natural Resin Resina Draconis to 3D Functionalized Fibrous Scaffolds for Efficient Chronic Wound Healing**

Shijie Guo^1^, Pengyu Wang^1,2^, Yu Sun^1^, Can Cao^1^, Junwei Gao^1^, Shihao Hong^1^, Ning Li^1,*^, Ruodan Xu^1,*^

1. *Department of Biomedical Engineering and Technology, Institute of Basic Theory for Chinese Medicine, China Academy of Chinese Medical Sciences, Beijing, China.*
2. *Guang’anmen Hospital, China Academy of Chinese Medical Sciences, Beijing, 100053, China.*

**^*^ Correspondence should be addressed to:**

Ruodan Xu, E-mail: xurd@ibtcm.ac.cn

Ning Li, E-mail: lin@ibtcm.ac.cn

**KEYWORDS:** Chronic wound healing; bioactive wound dressing; Resina Draconis; three-dimensional structure; wet electrospinning

**Figures**


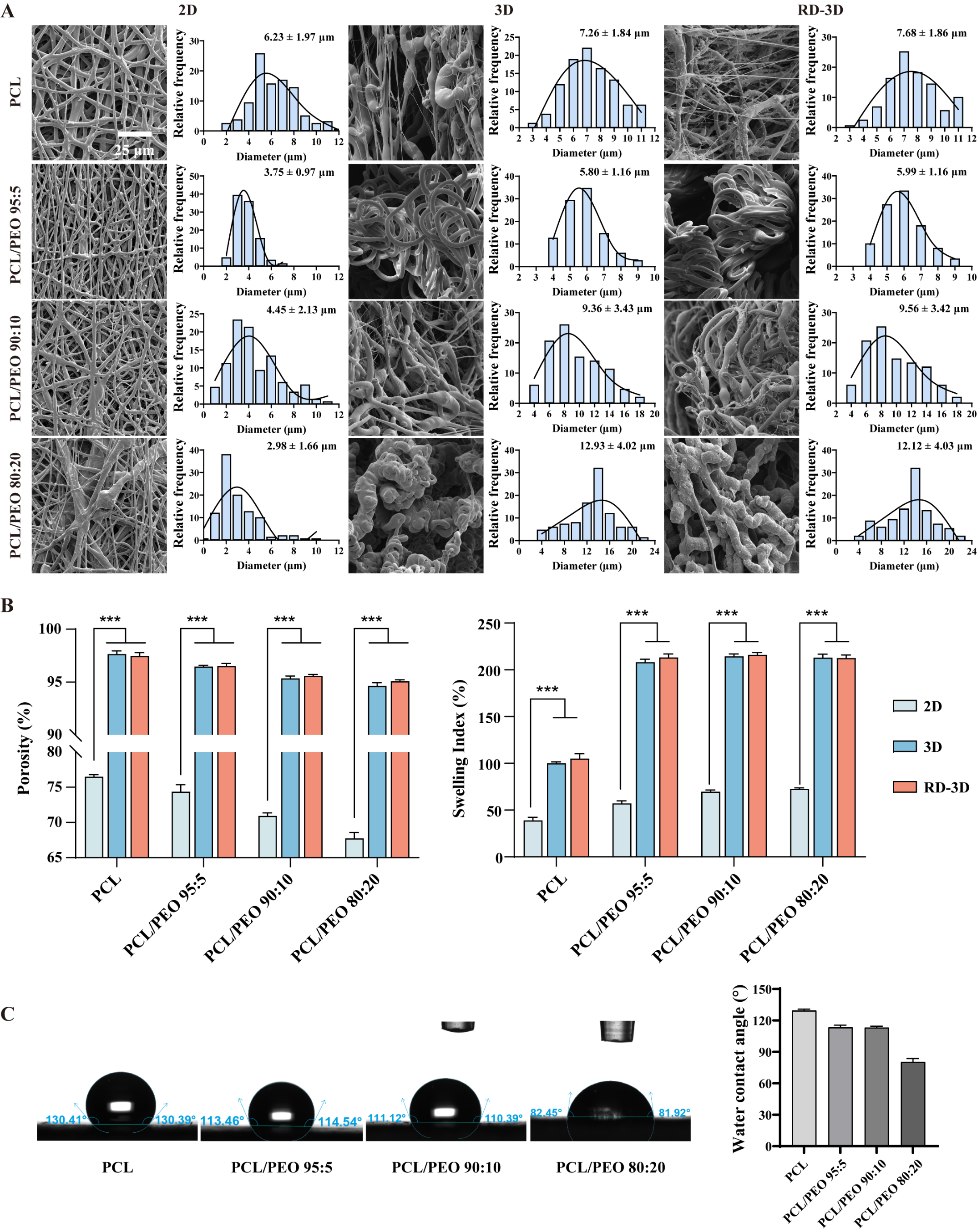


**Figure S1. Characterizations of fibrous dressings fabricated with different PCL/PEO ratios.** (A) SEM images (left) and corresponding diameter distributions (right) of the surface of PCL/PEO fibrous dressings fabricated with different PCL/PEO ratios, as indicated. Scale bar: 25 μm. (B) Porosity (left) and swelling index (right) of PCL/PEO fibrous dressings. *** *p*<0.005 *vs* 2D. (C) Images and quantifications of water contact angles of 2D with different polymer ratios. Bars represent mean ± SEM of 3 independent experiments.


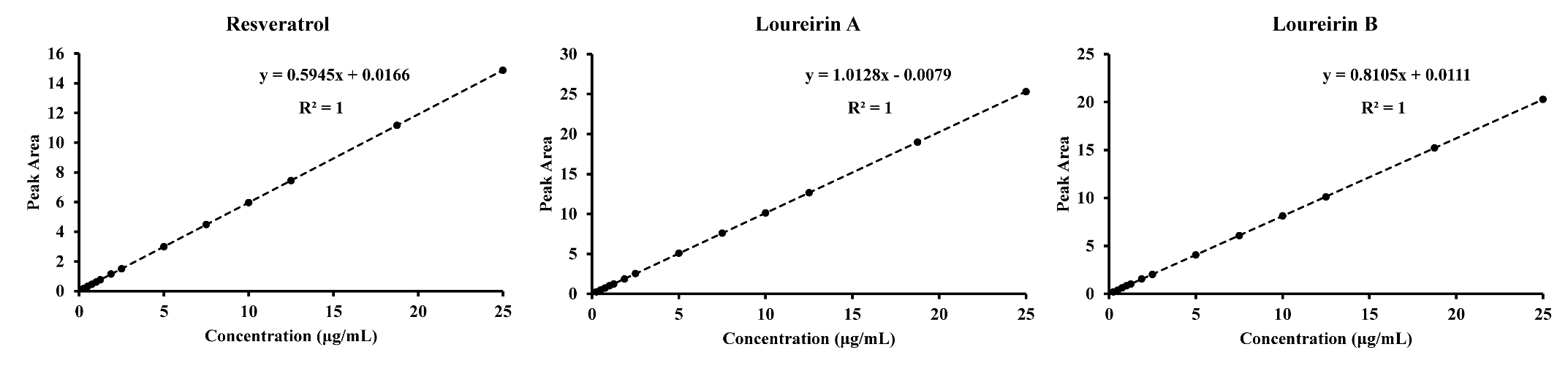


**Figure S2. Calibration curves for resveratrol, loureirin A and loureirin B.**


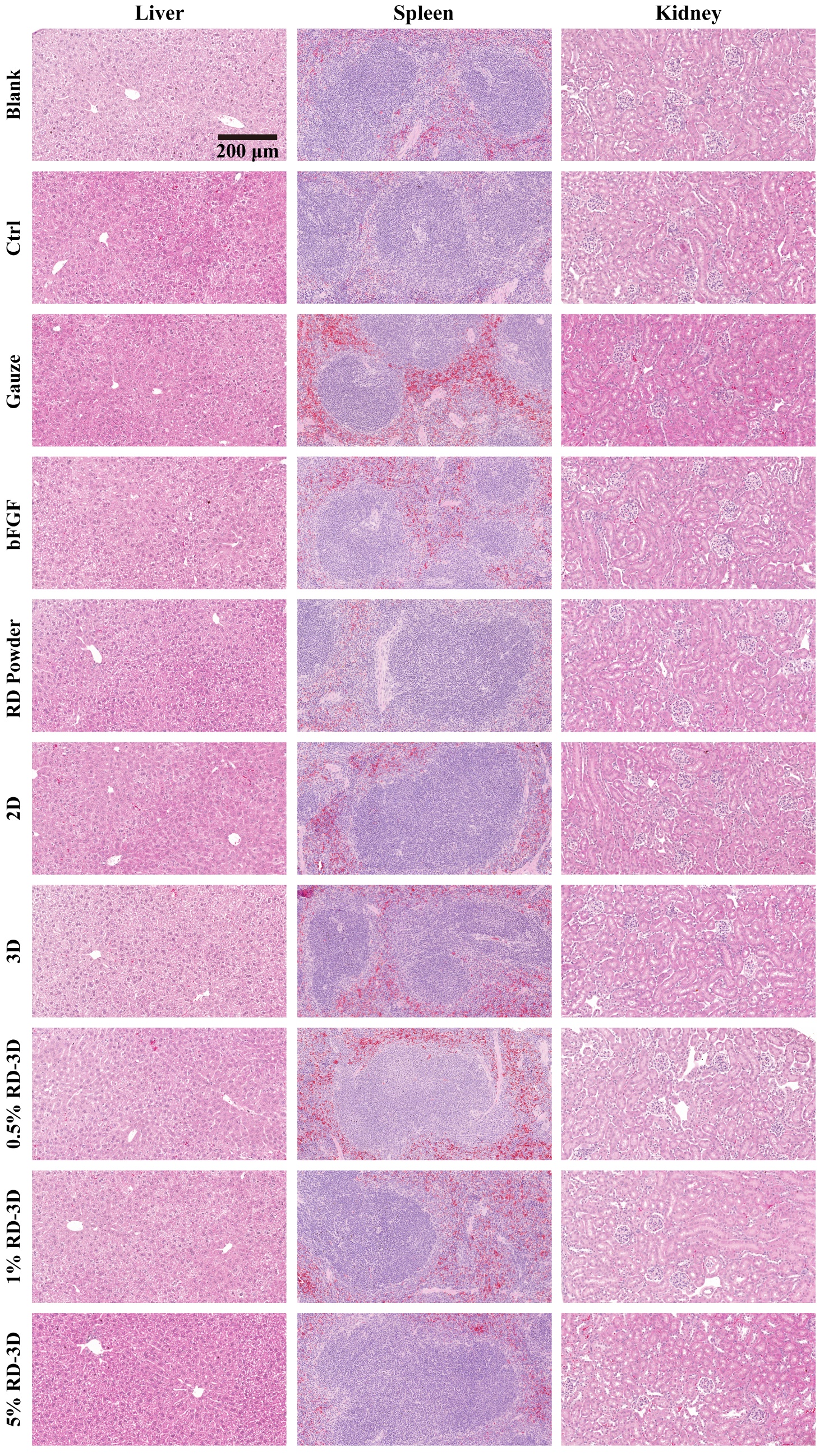


**Figure S3. *In vivo* assessment of the safety of PCL/PEO fibrous dressings in mouse pressure ulcer models.** H&E staining images of liver, spleen, and kidney for each group on day 8. Scale bar: 200 μm.

**
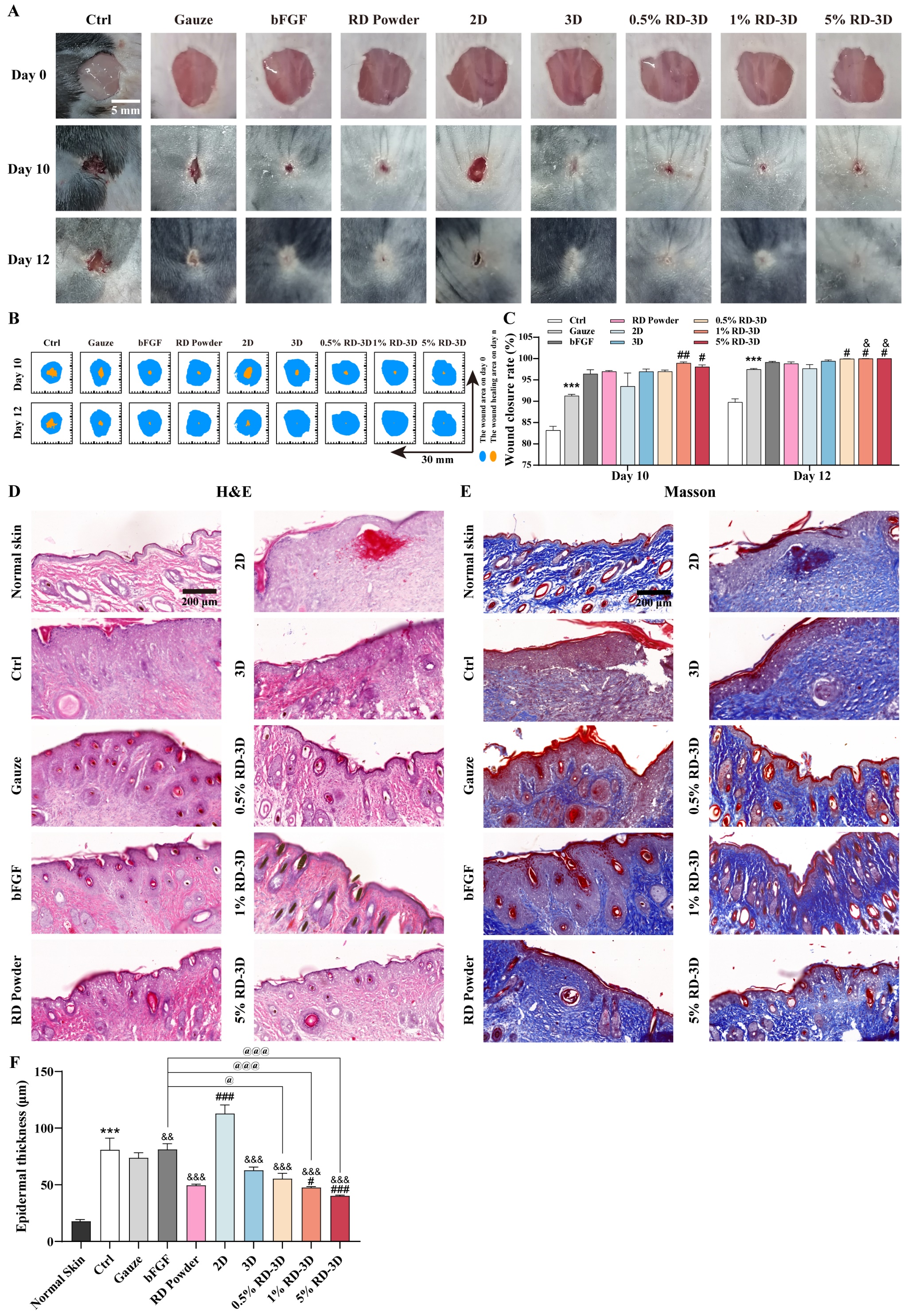
**

**Figure S4. Accelerated healing of infected wounds with RD-3D dressing over 12-days.** (A) Images of *S. aureus-*infected wounds treated by Gauze, recombinant bovine bFGF, RD powder, 2D, 3D, 0.5% RD-3D, 1% RD-3D, and 5% RD-3D, on day 0, 10, and 12, respectively. Scale bar: 3 mm. (B) Traces of wound closure on day 0, 10, and 12. (C) Quantification of wound size, n=3. *** *p<*0.005 *vs* Ctrl; # *p<*0.05, ## *p<*0.01 *vs* Gauze; & *p*<0.05 *vs* 2D. (D) H&E staining images of wound sections on day 12. Scale bar: 200 μm. (E) Masson’s trichrome staining images of wound sections on day 12. Scale bar: 200 μm. (F) Epidermal thickness was quantified according to pathological images (n = 3). *** *p<*0.005 *vs* Normal Skin; # *p<*0.05, ### *p<*0.005 *vs* Gauze; && *p*<0.01, &&& *p*<0.005 *vs* 2D; @ *p*<0.05, @@@ *p*<0.005 as indicated. Data are presented as the mean ± SEM.


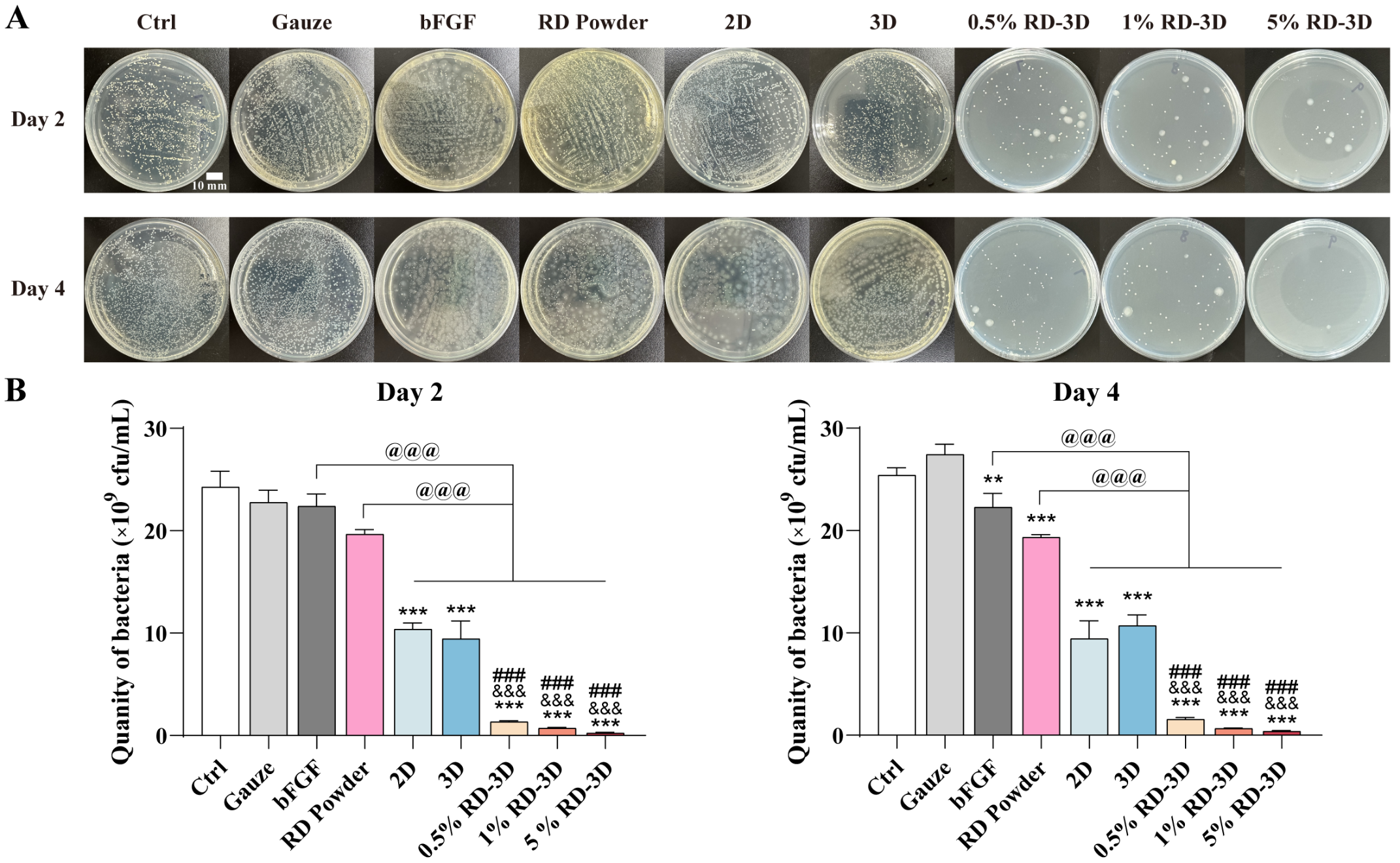


**Figure S5. *In vivo* antibacterial capacities of the administrations.** (A) Images of bacterial colonies derived from LB-cultured dressings at the end of the first and second administrations, representing wound infection on day 2 (top panels) and day 4 (bottom panels), respectively. Scale bar: 10 mm. (B) Quantification of bacterial colonies from (A), n=6. ** *p*<0.01, *** *p*<0.005 *vs* Gauze; &&& *p*<0.005 *vs* 2D; ### *p*<0.005 *vs* 3D; @@@ *p*<0.005 as indicated. Data are presented as mean ± SEM.

**
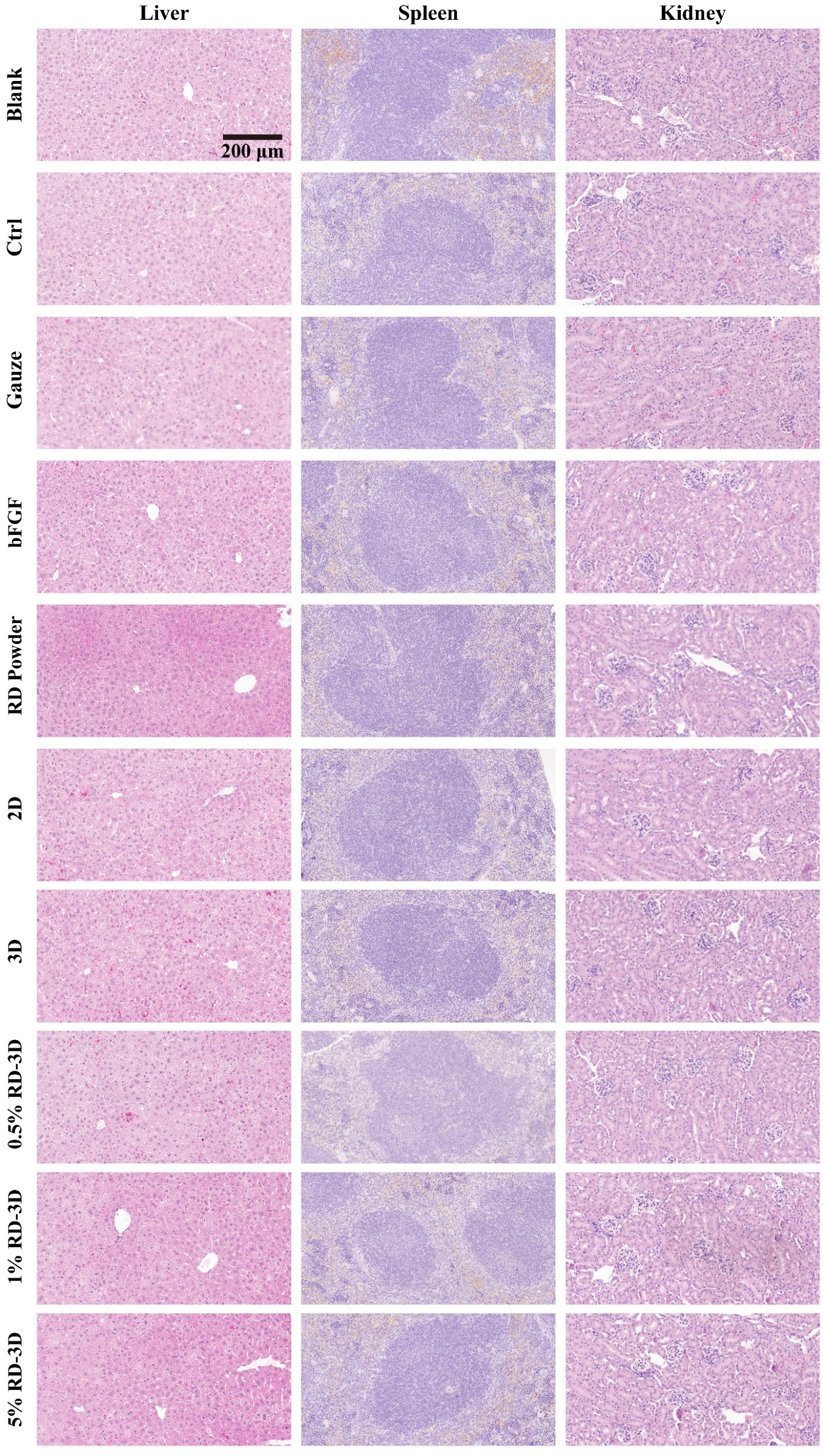
**

**Figure S6. *In vivo* assessment of the safety of PCL/PEO fibrous dressings in mouse *S. aureus-*infected wound models over 6 days.** H&E staining images of liver, spleen, and kidney for each group on day 6. Scale bar: 200 μm.


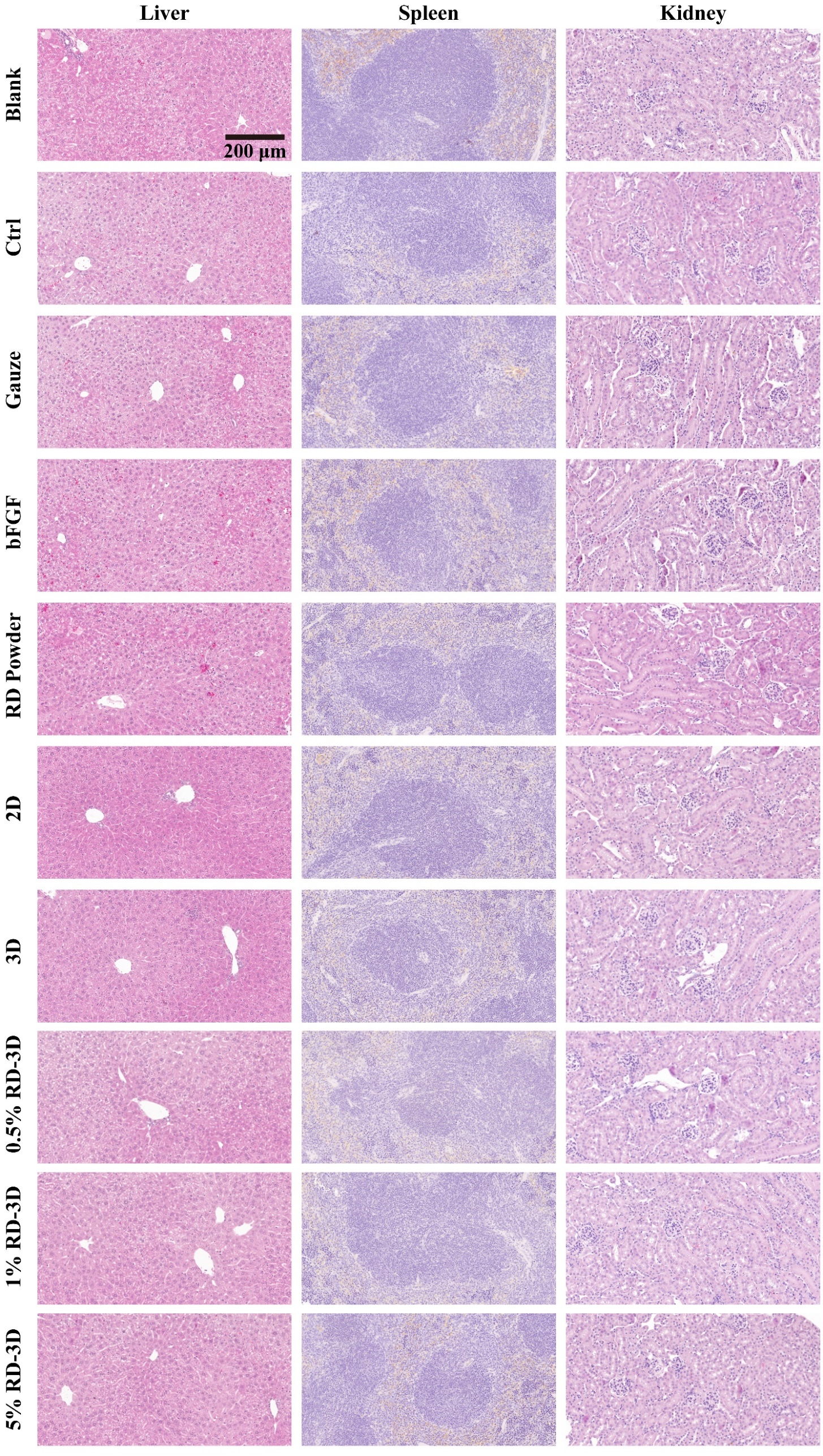


**Figure S7. *In vivo* assessment of the safety of PCL/PEO fibrous dressings in mouse *S. aureus-*infected wound models over 12 days.** H&E staining images of liver, spleen, and kidney for each group on day 12. Scale bar: 200 μm.

**
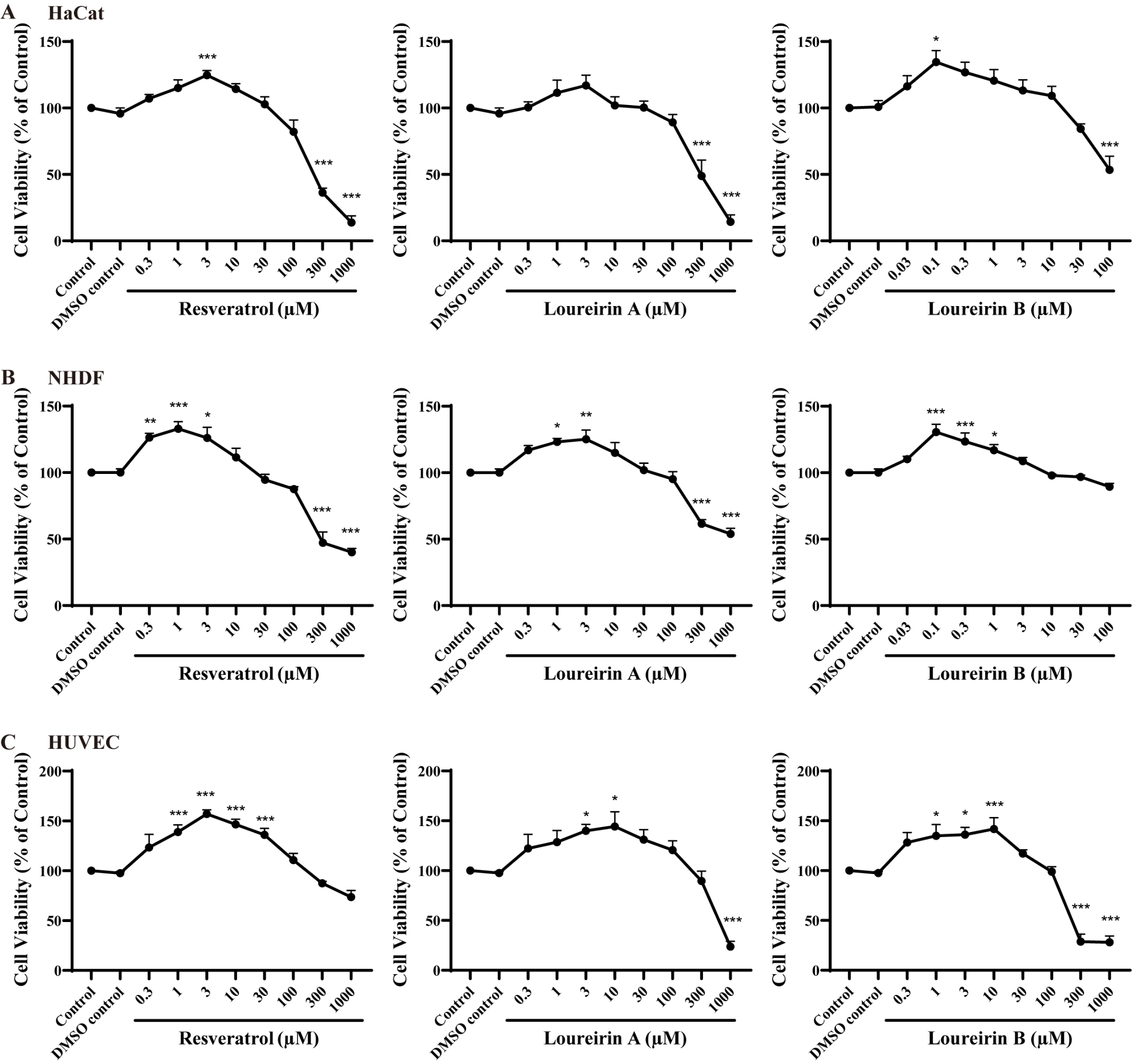
**

**Figure S8.** **Cytotoxicity of resveratrol, loureirin A, and loureirin B in experimental cells.** Cytotoxic responses of HaCat (A), NHDF (B), and HUVEC (C) cells to different concentrations of resveratrol (left), loureirin A (middle), and loureirin B (right) 24 h after treatments. * *p*<0.05, ** *p*<0.01, *** *p*<0.005 *vs* DMSO Control. Data are presented as the mean ± SEM of 3 independent experiments.

**Tables**

**Table S1. Electrospinning parameters for polymers at different ratios**

| **PCL/PEO Ratio (w/w)** | **Flow rate (mm/min)** | **Voltage (KV)** |
| --- | --- | --- |
| 100:0 | 0.15 | 8.5 |
| 95:5 | 0.15 | 9.5 |
| 90:10 | 0.15 | 10.5 |
| 80:20 | 0.12 | 12 |

**Table S2. The wavenumber of characteristic peaks for different samples**

| **Samples** | **Wavenumber (cm^-1^)** | **Functional group** |
| --- | --- | --- |
| PCL | 2945, 2868 | -CH_2_- |
|  | 1724 | C=O |
| PEO | 2880 | -CH_2_- |
|  | 1145, 1095, 1060 | C-O-C |
|  | 960 | C-O |
|  | 840 | C-H |
| 3D | 2945, 2880 | -CH_2_- |
|  | 1724 | C=O |
|  | 1145, 1095, 1060 | C-O-C |
|  | 960 | C-O |
|  | 840 | C-H |
| RD | 2950, 2870 | -CH_2_- |
|  | 1600, 1512, 1450 | benzene ring |
| RD-3D | 2949, 2868 | -CH_2_- |
|  | 1726 | C=O |
|  | 1608,1516, 1471 | benzene ring |
|  | 1170, 1100, 1068 | C-O-C |
|  | 962 | C-O |
|  | 842 | C-H |
